# A pan-cancer transcriptomic study showing tumor specific alterations in central metabolism

**DOI:** 10.1038/s41598-021-93003-3

**Published:** 2021-07-01

**Authors:** Ilir Sheraj, N. Tulin Guray, Sreeparna Banerjee

**Affiliations:** 1 Department of Biological Sciences, Orta Dogu Teknik Universitesi (ODTU/METU), Ankara, 06800 Turkey; 2 Cancer Systems Biology Laboratory (CanSyl), Orta Dogu Teknik Universitesi (ODTU/METU), Ankara, 06800 Turkey

**Keywords:** Data mining, Multienzyme complexes, Enzyme mechanisms

## Abstract

Recently, there has been a resurgence of interest in metabolic rewiring of tumors to identify clinically relevant genes. However, most of these studies have had either focused on individual tumors, or are too general, providing a broad outlook on overall changes. In this study, we have first curated an extensive list of genes encoding metabolic enzymes and metabolite transporters relevant to carbohydrate, fatty acid and amino acid oxidation and biosynthesis. Next, we have used publicly available transcriptomic data for 20 different tumor types from The Cancer Genome Atlas Network (TCGA) and focused on differential expression of these genes between tumor and adjacent normal tissue. Our study revealed major transcriptional alterations in genes that are involved in central metabolism. Most tumors exhibit upregulation in carbohydrate and amino acid transporters, increased glycolysis and pentose phosphate pathway, and decreased fatty acid and amino acid oxidation. On the other hand, the expression of genes of the tricarboxylic acid cycle, anaplerotic reactions and electron transport chain differed between tumors. Although most transcriptomic alterations were conserved across many tumor types suggesting the initiation of common regulatory programs, expression changes unique to specific tumors were also identified, which can provide gene expression fingerprints as potential biomarkers or drug targets. Our study also emphasizes the value of transcriptomic data in the deeper understanding of metabolic changes in diseases.

## Introduction

Rewiring of cellular metabolism to aid the initiation and progression of tumors has come to be recognized as a hallmark of cancers^1^. All tumors, irrespective of their origin show increased levels of glycolysis despite the presence of oxygen. The increased utilization of glucose in tumor cells is a feature that is routinely exploited to detect and visualize them by fluorodeoxyglucose positron emission tomography (FDG-PET) scans^2^. Metabolic changes in cancer were first recognized by Otto Warburg in a seminal study in 1926 in which he proposed that in contrast to non-transformed cells, cancer cells rely on aerobic glycolysis (Warburg effect) for their energy needs as a result of impaired mitochondrial function^3^. Altered metabolism in cancer cells gained attention after Warburg’s proposal; however, interest in the field waned after his death aided by a subsequent rejection of the mitochondrial dysfunction hypothesis^4^. Additionally, the discovery of oncogenes backed by an increasing interest in sequencing technologies brought forth the somatic mutation theory in full force. Scientists aimed to identify recurring mutations across all cancer types and develop drugs to target them^5^. However, despite the massive amount of genomic data generated across different tumor types, the benefit of this approach was limited due to the inherent heterogeneity of tumors and the broad spectrum of somatic mutations^6,7^. Thus, the need for new approaches became apparent and a modified version of Warburg’s theory gained attention according to which metabolic reconstitution under the control of oncogenes is an essential process for cancer growth and survival^8^. Currently, about 250 oncogenes and 700 tumor suppressor genes have been identified, the majority of them playing important roles in controlling key metabolic pathways in tumors^9^. Additionally, widespread transcriptional dysregulation of metabolic genes in cancer have been reported^10^ with major metabolic enzymes regulating oncogenic signaling^11^.

Several large scale studies have used transcriptomics to compare metabolic pathways between cancer and adjacent normal tissues or between different cancer types. Gaude and Frezza reported a general deregulation of mitochondrial function with widespread downregulation of genes involved in oxidative phosphorylation (OXPHOS) across multiple tumor types, a characteristic that was correlated with the upregulation of genes associated with epithelial-to-mesenchymal transition (EMT) and poor patient prognosis^10^. Hu et al., also studied the changes in expression between tumors and their adjacent normal samples across 22 cancers and found each tumor’s metabolic programs to be more similar to its parental tissues than to other tumors. In addition, these authors also reported tumor-specific expression differences for multiple isoforms of the same enzyme, especially those involved in carbohydrate metabolism^12^⁠. More recently, RNA sequencing from TCGA was used to study the changes in metabolic genes across 33 tumor types and classify them according to 7 major metabolic pathways. The study showed that tumors with upregulated carbohydrate, nucleotide and vitamin metabolism were associated with poor prognosis, while those with upregulated beta oxidation had better prognosis. Amino acid metabolism and energy integration pathways on the other hand showed mixed clinical results^13^. In another study, 26 tumors and their matched normal samples were compared for changes across 114 metabolic pathways. This pipeline was able to identify metabolic pathways that were broadly dysregulated across tumors as well as within the same tumor. In addition, the authors reported that Master Metabolic Transcriptional Regulators (MMTR) can serve as biomarkers that can predict the outcome of therapeutics directed against metabolism-related genes^14^. All the above-mentioned studies take a systemic approach at comparing the differences between tumors and their adjacent normal tissues or across multiple tumors. However, systemic approaches in understanding metabolism may suffer from at least two drawbacks; the high pathway inter-connectivity, which leads to over-representation of a number of genes, and the current indiscriminate nature of the approach of assigning equal weights to all genes in the network. Some authors have used various statistical approaches to deal with the problem of interconnectivity^10^ but the second drawback still persists, although it is a well-known fact that the flux through metabolic pathways is heavily determined by the expression and/or activity of rate-limiting enzymes.

**Figure 1:**
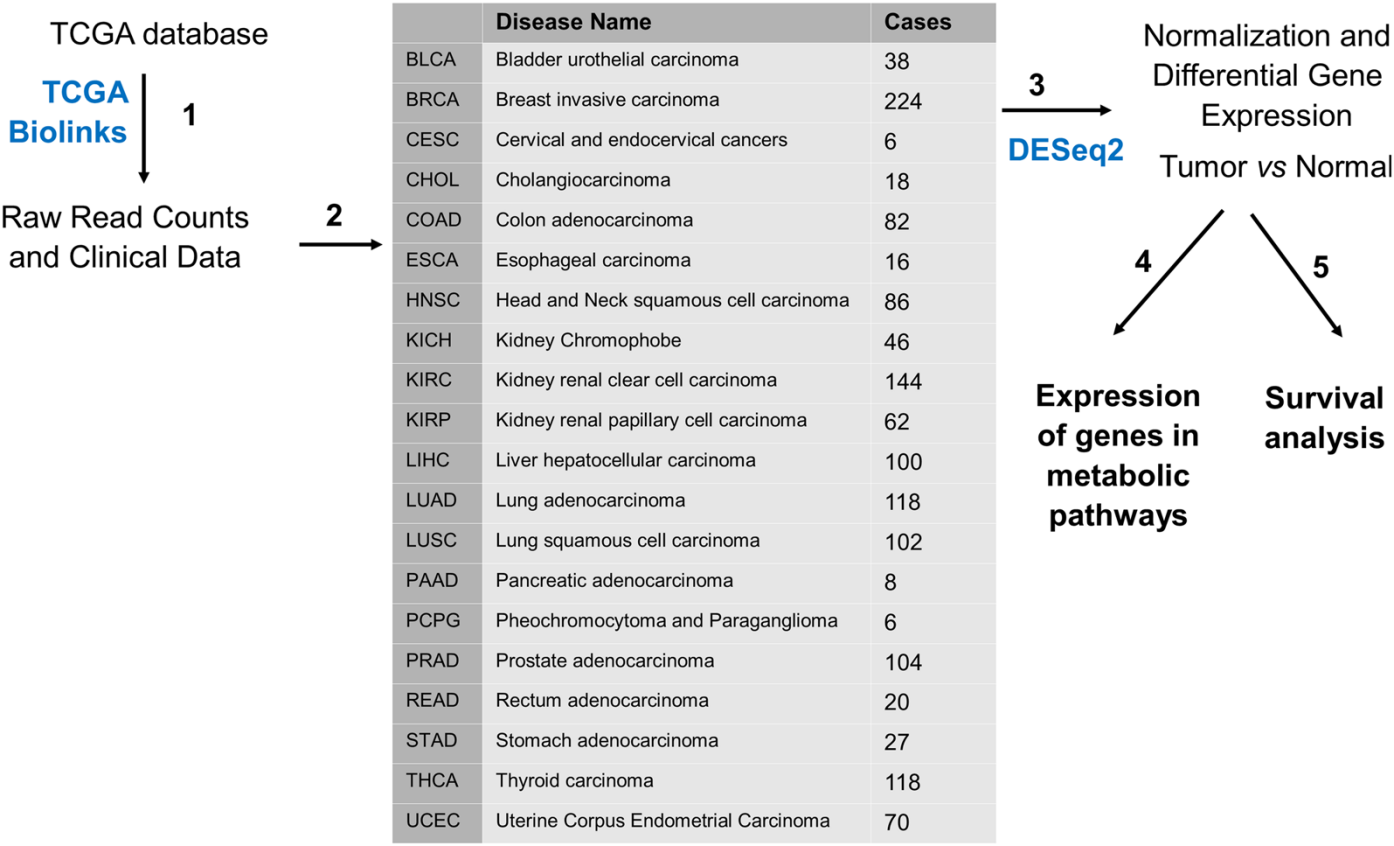
Summary of the pipeline used for analysis. (Step 1) Clinical information and raw read counts in HTSeq format were downloaded for each cohort from TCGA database with the *TCGABiolinks* package. (Step 2) Tumors were matched with their adjacent normal tissues. Any cohort having less than three matched normal samples per tumor was excluded from the analysis. Table shows cohort code, the name of each tumor and the total number of samples analyzed for each. (Step 3) *DESeq2* package was used for normalization and differential gene expression for each cohort. (Step 4) Log-fold change values and statistical significance of target genes were extracted from the expression matrix and used for heat map construction (Step 5). Based on the results of step 4, correlations of certain genes with patients’ survival were analyzed.

In the current study, we analyzed RNA sequencing data from TCGA to compare differential gene expression (DGE) for 20 tumors and their adjacent normal tissues (Fig. 1). In total, 1386 samples were processed, half of them tumor and the other half healthy adjacent tissues. Unlike the studies mentioned above, we have taken a more targeted approach and have analyzed the expression of genes related specifically to the transport of macromolecules into cells and their metabolism. By manually curating the pathways, we have assigned each gene to a specific metabolic pathway to avoid redundancy. Additionally, by using simple visualizations we show that the expression of each enzyme in the pathway is independent of others. To our knowledge, this is the first study that comprehensively evaluates the expression of sugar and amino acid transporters in the cell membrane and mitochondria. Keeping with our focus on dysregulation of tumor bioenergetics, expression of genes involved in carbohydrate, fatty acid, ketone bodies, amino acid, Krebs Cycle (TCA), anaplerosis and OXPHOS pathways were also evaluated. Since we manually curated all the genes encoding enzymes and their isoforms from the published literature, we were able to include genes that were not present in databases; additionally, we removed some genes that were irrelevant to the specific pathways in question. This approach enabled us to observe not only the expression of genes that were dysregulated across many tumor types, but also those that were unique to certain tumors and had a great impact on patient survival. In broad terms, our study supports the existing literature and shows that tumors rely heavily on carbohydrate and amino acid influx, the former to be used in glycolysis and pentose phosphate pathway (PPP), while the latter is mostly preserved for protein synthesis and oxidative stress mitigation in highly hypoxic tumor environments. We also demonstrate that most tumors display suppressed fatty acid beta oxidation (FAO) and elevated fatty acid biosynthesis (FAS), while genes in the TCA cycle and OXPHOS do not exhibit any predictable pattern of expression across different tumor types. Finally, in agreement with previous studies, we show that different tumors express different isoforms of the same enzyme, which can be selected and targeted for more effective treatments in the future.

## Results and discussion

### Carbohydrate metabolism in tumors

#### Deregulation of transporters

**Figure 2:**
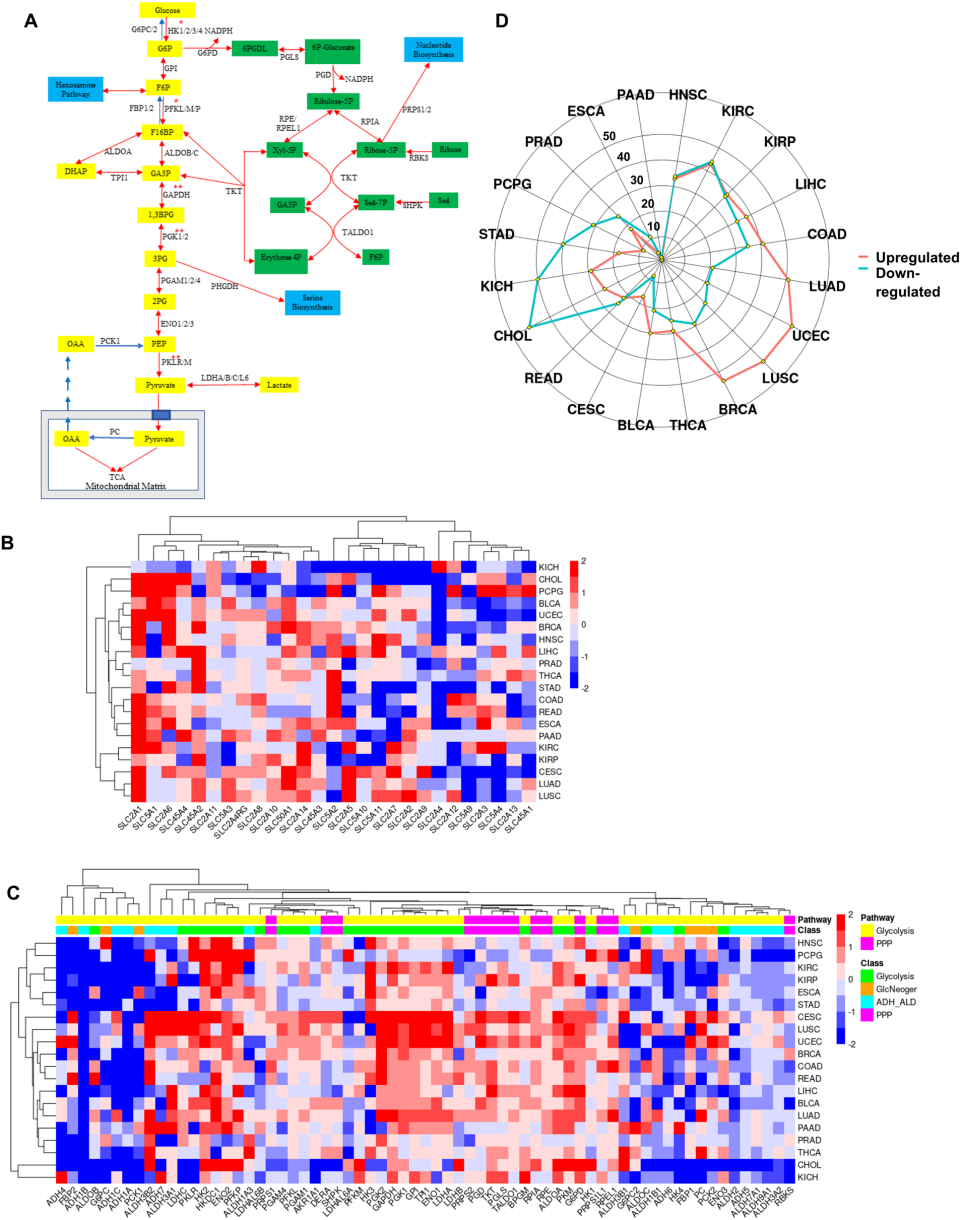
Alterations in enzymes of carbohydrate metabolism between cancer and matched normal samples. (**A**) A simplified schematic representation of glycolysis (yellow boxes), pentose phosphate pathway (PPP) (green boxes) and gluconeogenesis (reactions in blue arrows). The reactions in which ATP is utilized are annotated with single asterisks, those in which ATP is produced with 2 asterisks. Blue boxes represent pathways interconnected to central carbohydrate metabolism. (**B**) Heat map showing the expression of hexose transporters. For these genes the modern SLC nomenclature was used followed by family and class member. The color bar on the right represents the log-fold changes; red shows genes upregulated in tumors and blue shows genes downregulated in tumors as compared to normal samples. (**C**) Heat map showing the expression of genes for glycolysis and PPP. Pathway bar shows glycolytic (yellow) and PPP (pink) genes, while the class bar divides them into glycolysis (green), gluconeogenesis (orange), aldehyde and alcohol dehydrogenases (cyan) and PPP (pink). (**D**) Radar plot showing the total number of genes significantly (LFC > 0.5, FDR < 0.05) upregulated (red) or downregulated (blue) in tumors compared to normal tissue for each cohort. The full names of genes are given in Supplementary Tables S1 and S2.

An overview of glucose metabolism, incorporating glycolysis and pentose phosphate pathway (PPP) is shown in Fig. 2A. Glycolytic flux is known to be regulated at the steps of glucose entry via glucose transporters, the two committed steps of glycolysis [HK and Phosphofructokinase (PFK)] and the transport of lactate^15^⁠. Enhanced expression of hexose transporters is implicit in increased sugar utilization by cancer cells. DGE for a total of 26 genes covering the three main families of hexose transporters, namely SLC2, SLC5 and SLC45^16,17^ were evaluated (Fig. 2B). SLC2A1 (GLUT1) was highly expressed in all tumors except KICH and PRAD, while SLC2A4 (GLUT4) was downregulated in all tumor types except in KICH. No consistent expression pattern was observed for the other transporters. Downregulation of GLUT1 in PRAD can explain the difficulty in obtaining clear FGD-PET imaging in primary PRAD tumors^18^. Studies have shown that GLUT1 has a high affinity for glucose, galactose and mannose and this transporter is normally highly expressed in the blood–brain barrier where it determines the rate of glucose entry into the brain. High expression is also seen in erythrocytes, which rely exclusively on glycolysis for ATP generation; and the placenta, which has been shown to use glucose extensively with GLUT1-null mice showing embryonic lethality^19^. Survival analysis showed that high expression of GLUT1 was correlated with poor Overall Survival (OS) when patients were separated according to its median expression in LIHC (HR 1.9, p = 7e−04), LUAD (HR 1.7, p = 3e−04) and PAAD (HR 1.6, p = 0.02). These data suggest that GLUT1 could be a major driver of Warburg effect; therefore, developing drugs that can exclusively target the channel expressed in tumor cells, or reducing the amount of carbohydrate intake in the diet may improve cancer management and therapy. On the other hand, SLC2A2 (GLUT2) is the major sugar transporter in hepatic tissues and has a relatively low affinity for glucose. Our data show that it was downregulated in LIHC, a state that is associated with poor prognosis (HR 2.1, p = 6e−05). SLC2A3 (GLUT3) showed a mixed pattern of up- and downregulation in various tumors. SLC2A5 (GLUT 5) is the main transporter of fructose, and its expression was significantly upregulated in 7 tumors. LUAD is one such tumor where the upregulation of SLC2A5 was shown to enhance tumorigenicity; inhibition of SLC2A5 in lung cancer cell lines using the fructose analog 2,5-anhydro-d-mannitol sensitized the cells to paclitaxel^20^⁠. Some members of the SLC5 and SLC45 families showed significant upregulation in more than half of the tumors, with SLC45A2 showing a very strong upregulation in PRAD (Fig. 2B). This family of transporters is known to uptake disaccharides and shows increased absorption capacity in acidic environments, helping tumors to further increase sugar uptake for glycolysis^21^.

Pyruvate and lactate, the end products of glycolysis are transported across the plasma membrane via monocarboxylate transporters (MCT) 1–4^22^⁠. These proteins form a part of the larger SLC16 family of carrier proteins. MCT-4 is considered to be particularly suited for the export of lactate from highly glycolytic cells; additionally, MCT-4 has low affinity for pyruvate, suggesting this transporter to be particularly suited for cancer cells that are known to exhibit high NAD+/NADH ratio^23^⁠. Indeed, we observed that the expression of MCT4 was the highest among the four lactate transporters (Supplementary Fig. S1) and high expression of MCT4 was associated with poor prognosis in BLCA (HR 1.43, p = 0.02), CESC (HR 2.0, p = 0.01), LIHC (HR 1.9, p = 6e−4) and LUAD (HR 1.83, p = 8e−5), corroborating previous data^24^⁠. MCT-4 is also the transporhat was identified to regulate glycolytic flux^15^⁠.

#### Carbohydrate metabolism: glycolysis, PPP and hexosamine biosynthesis pathway

We next analyzed the expression of glycolysis, pentose phosphate pathway (PPP) and the hexosamine biosynthesis pathway (HBP) (Supplementary Fig. S2) genes in the different tumor types. These three pathways cover all possible fates of carbohydrates once they are transported into the cell. mRNA expression of glycolytic enzymes was strongly upregulated in all tumor types, as expected (Fig. 2C). Phosphorylation of glucose by hexokinase (HK) and fructose-6-phosphate by Phosphofructokinase (PFK) are considered to be the two committed steps of glycolysis that can regulate the flux of metabolites through the pathway^15^⁠. HK is known to be expressed as four isoforms, HK1-4. We observed that HK2 was significantly overexpressed in 16 out of the 20 tumors, and only significantly downregulated in COAD. In addition, its higher expression was associated with poor OS in LIHC (HR 1.5, p = 0.03) and CESC (HR 2.1, p = 0.008). HK4 is known to be mostly expressed in the liver and kinetic studies have shown that it has a comparatively lower affinity for glucose^25^. On the other hand, HK2 has about 250-fold higher affinity for glucose compared to HK4^26^ and it has been shown to directly interact with a voltage-dependent anion channel (VDAC1) on the outer mitochondrial membrane of tumor cells. This interaction gives HK2 preferential access to ATP for glucose phosphorylation and at the same time competitively prevents the interaction of VDAC1 with pro-apoptotic proteins, preventing cellular apoptosis^27–29^. We found HK3 to be upregulated in half of the tumors and showed significant correlation with poor OS in KIRC (HR 1.9, p = 2e−05), while HK1 was significantly upregulated in only 3 tumors (PCPG, CHOL and CESC).

PFK-1 catalyzes the second committed step of glycolysis converting fructose 6-phosphate to fructose 1,6-bisphosphate using ATP. PFK1 exists in three isoforms in mammals, PFK-M (expressed in muscle tissue), PFK-P (found in the plasma) and PFK-L (expressed in the liver)^30^⁠. PFK-P is expressed in most tissues and can be allosterically regulated by ATP (inhibition) and fructose 2,6-bisphosphate (activation)^31^⁠. We observed increased expression of PFK-L and PFK-P several different cancer types with particularly high expression of PFK-P in LUAD, LUSC, KIRC, LIHC and CHOL (Fig. 2C, Supplementary Fig. S1). Pyruvate Kinase isoenzyme M (PKM) catalyzes the transfer of a phosphate from phosphoenolpyruvate (PEP) to ADP thereby generating ATP is the last step of glycolysis. This enzyme is allosterically activated by Fructose-1,6-bisphosphate^32^⁠. A remarkable concordance in the expression of PFK-P and PKM was observed suggesting common regulatory pathways that can allow glycolysis to be active when nutrients are available^31^⁠. The expression of PFK-M did not change dramatically, which is expected since this isoform is primarily known to be expressed in skeletal muscle^30^⁠.

We observed increased expression of a little known and often overlooked enzyme called hexokinase domain containing 1 (HKDC1) in nearly half of the tumors (Fig. 2C). Kinetic studies have shown that this enzyme has low affinity for glucose and is considered to be the fifth member of the HK family^33^. Though the high expression of HKDC1 in LIHC was associated with poor overall survival (OS), this has not been addressed mechanistically yet^34^. Another recent study showed that high expression of HKDC1 was also associated with poor prognosis in LUAD by inducing EMT and activation of mTOR pathway^35^. Future studies are needed to evaluate the role and importance of this gene in tumorigenesis. The expression of the remaining glycolytic enzymes was increased in all tumors, with different isoforms showing different patterns of expression (Fig. 2C). An interesting exception was PRAD, which showed very mild changes in glycolysis, an observation that corroborates our data (Please see the section FAO) as well as previous studies showing that PRAD relies primarily on FAO for the generation of energy^36^.

The cofactor NADPH functions as an electron carrier for enzymatic reactions that maintain redox balance and generate biomass in cells. Deuterium isotope studies have been utilized to establish the strict compartmentalization of cytosolic and mitochondrial NADPH. This allows the maintenance of distinct NADPH/NADP+ ratios in these subcellular locations, thereby enabling location-specific metabolic processes^37^⁠. Classically, the PPP is one of the primary sources of cellular NADPH with malic enzyme and serine-driven one-carbon metabolism mediated by tetrahydrofolate also contributing towards to cellular NADPH pool^38^⁠. NADPH serves as the reducing currency for most biosynthetic reactions and protects cells from oxidative stress by reducing oxidized glutathione^39^. The activity of this pathway is therefore expected to be high in cancer cells. PPP is divided into two branches, the oxidative and the non-oxidative branch. The oxidative branch oxidizes glucose 6-phosphate via the rate-limiting enzyme glucose-6-phosphate dehydrogenase (G6PD) and 6-phosphogluconate dehydrogenase (PGD), with both reactions generating NADPH. The non-oxidative branch is catalyzed by TKT and TALDO1, and it reconnects the oxidative branch to glycolysis (Fig. 2A). While the oxidative branch is essentially irreversible, the non-oxidative branch is reversible, depending on the stoichiometry of the metabolites in the specific cellular context. The end product of the non-oxidative branch is ribulose 5-phosphate (RB5P) that is either utilized for nucleotide biosynthesis, or is returned to glycolysis via TKT and TALDO1^39^_._ We observed that the expression of G6PD was significantly increased in 16 tumors, 10 of them very strongly (> 1 log-fold change (LFC)), a state that was associated with poor OS in 3 tumors; KIRC (HR 1.8, p = 3e−04), KIRP (HR 2.4, p = 1e−03) and LIHC (HR 1.9, p = 3e−04). None of the tumors showed any significant downregulation of G6PD (Fig. 2C). TKT and TALDO1 on the other hand were significantly upregulated in some tumors but not as consistently as G6PD, showing that oxidative branch of PPP is most likely essential for cancer development.

The HBP branches from glycolysis after the formation of fructose 6-phosphate (Supplementary Fig. S2A). Although generally overlooked in the context of cancer metabolism, the HBP is important for many reasons. First, this pathway is at the crossroads of metabolism of all macromolecules in the cell: amino acid via glutamine/glutamic acid, fatty acid via acetyl-CoA, carbohydrates via fructose as it branches off and also feeds into glycolysis via Leloir pathway, and nucleic acid via uridine-5′-triphosphate (UTP) (Supplementary Fig. S2A). It is therefore not surprising that HBP has been suggested as an energy sensor of the cell^40^. In addition, UDP-N-acetyl glucosamine (UDP-GlcNAc), the end product of this pathway, is used for N- and O-glycosylation of proteins, a process that is deregulated in many tumors^40^. The rate limiting step of HBP is catalyzed by glutamine-fructose-6-phosphate transaminase 1/2 (GFPT1/2) by using glutamine as a co-substrate while the reverse reaction is catalyzed by glucosamine-6-phosphate deaminase 1/2 (GNPDA1/2). Our results show an overall pattern of increased expression of the HBP enzymes in many tumors; however, none of the genes were upregulated as strongly as the enzymes of glycolysis and most of the upregulations did not reach statistical significance (Supplementary Fig. S2B). Of interest, the overexpression of O-GlcNAcylation transferase (OGT1) was significantly upregulated in COAD and READ, and a recent study showed that high expression of this enzyme enhanced colon cancer metastasis and was associated with poor prognosis^41^. We also observed a general downregulation of enzymes of the Leloir pathway of galactose metabolism. This pathway has not been examined in most tumor types. Tang et al., reported that knockdown of GALK1 and GALT in HepG2 liver cancer cells resulted in the inhibition of cellular growth, implying an oncogenic function of this pathway^42^. However, these authors did not report any mechanistic details and according to our results, GALK1 did not show any significant upregulation in LIHC.

Another pathway by which glucose can be metabolized is the polyol pathway. This pathway consists of alcohol dehydrogenases (ADHs) that can oxidize alcohols such as ethanol into acetaldehydes; the latter are further oxidized into ketones by aldehyde dehydrogenases (ALDHs). The ADH and ALDH family of enzymes have 7 and 19 known members, respectively, and some of them are functional in the cytosol while others in the mitochondria^43^. The polyol pathway is involved in detoxification of aldehydes such as acetaldehyde and induces differentiation of epithelial cells via the retinal metabolic pathway^44^. They also contribute to cellular bioenergetics by reducing NAD^+^ to NADH, which is then used to generate ATP in mitochondria. We observed that most tumors showed a decrease in expression of both ALDHs and ADHs (Fig. 2C). Elevated aerobic glycolysis in most tumor cells leads to increased cytosolic NADH levels, and the resulting low NAD^+^/NADH ratio may be responsible for suppressing the expression of ADH and ALDH by mass-action. Although not all members of ADH/ALDH families were downregulated in all tumors, this may also be the result of coenzyme preferences, as some of them prefer NADP^+^ instead of NAD^+^^43^. Recently, a study showed that blocking the enzymatic activities of all ALDHs in LUSC led to decreased levels of NADH, causing cell death^45^. This however does not contradict our model as many of ALDHs such as ALDH1L1 and ALDH18A1, which have the greatest impact on ATP levels are found in the mitochondria, therefore their activities are not affected by high levels of NADH in the cytosol.

Gluconeogenesis results in the synthesis of glucose and uses most of the enzymatic reactions of glycolysis with the exception of the four irreversible steps: carboxylation of pyruvate into oxaloacetate (OAA) by pyruvate carboxylase (PC); conversion of OAA into Phosphoenolpyruvate (PEP) by PCK1/2, conversion of Fructose 1,6-Bisphosphate (F16BP) into Fructose 6-Phosphate (F6P) by FBP1/2, and dephosphorylation of Glucose 6-Phosphate (G6P) to Glucose by G6PC (Fig. 2A)^46^. Gluconeogenesis takes place mostly in the liver, which provides glucose to organs such as the brain during long periods of fasting. Kidneys, muscle and intestine are some of the other organs where gluconeogenesis also takes place^47^. DGE in the current study showed that the pathway was significantly downregulated in all tumors (Fig. 2C), especially LIHC, COAD and KIRC; however, the expression of PC was higher in LUAD, LUSC and THCA compared to their normal counterparts. This is in agreement with a recent study where it was shown that in LUSC cell lines, PC acts as an anaplerotic enzyme converting pyruvate into OAA inside the mitochondrial matrix to be fed into the TCA, in this way increasing FAS and promoting cell cycle progression^48^. Overall, more than half of the tumors, especially LUAD, LUSC, UCEC and BRCA, had more significantly upregulated genes involved in carbohydrate metabolism and transport as compared to downregulated ones, while for some, such as CHOL, KICH STAD and PCPG, the number of downregulated genes was higher (Fig. 2D). The rest did not show significant changes, while PAAD and ESCA were exceptions with very little changes, probably due to very high heterogeneity of samples (Supplementary Fig. S3).

### Fatty acid metabolism

The importance of fatty acid (FA) metabolism in cancer progression, survival and metastasis has received increasing attention in recent years^49,50^. FAs are essential for many cellular processes. They are the constituents of cell membranes, the composition of which is of major importance in cellular physiology. Additionally, FAs are involved in cellular signaling, which regulates a myriad of cellular processes, especially growth and metastasis; and their oxidation produces ample amounts of energy for the cell^51^. Keeping in mind the highly complicated and interconnected processes of FA metabolism, we have separately evaluated the expression of genes for fatty acid oxidation (FAO), incorporating β-oxidation and ketone body metabolism, and fatty acid synthesis (FAS), incorporating cellular FAS (FAS-I) and mitochondrial FAS (FAS-II).

#### β-oxidation and ketone body metabolism

**Figure 3:**
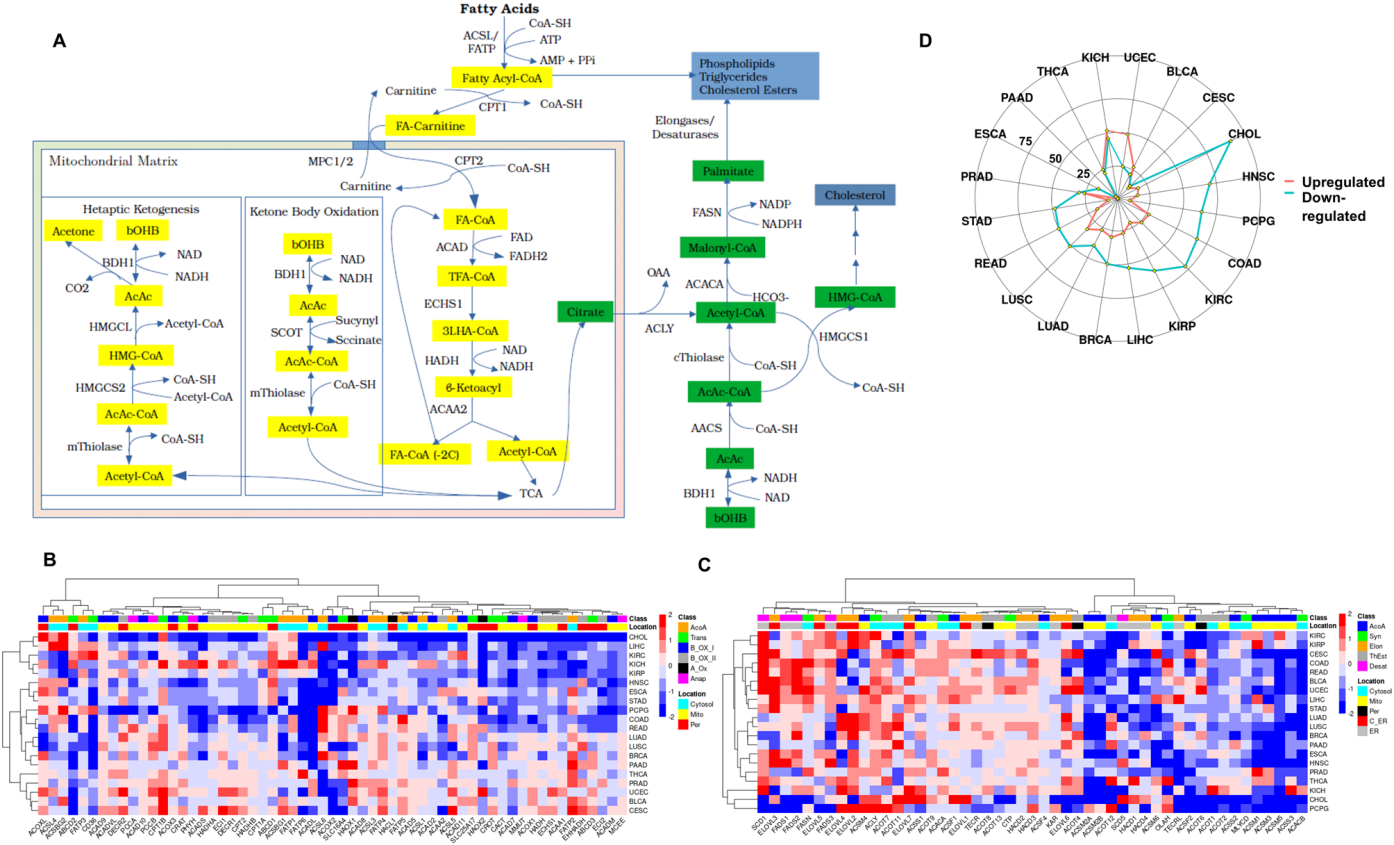
Alterations in enzymes of lipid metabolism between cancer and matched normal samples: (**A**) A simplified schematic representation of fatty acid metabolism. Beta oxidation (yellow boxes) starts with the esterification of coenzyme A (CoA) to the fatty acid, transportation of fatty acyl-CoAs into the mitochondrial matrix and their oxidation into acetyl-CoA. Fatty acid synthesis intermediates are shown in green boxes, hepatic ketogenesis and extrahepatic ketone body oxidation are enclosed in separate boxes. (**B**) Heat map showing the expression of genes involved in fatty acid oxidation. Class color bar shows Acetyl-CoA transferases (orange), Carnitine system (green), first step of beta oxidation by ACADs (blue), the remaining steps of beta oxidation (gray), alpha oxidation (black) and a number of enzymes involved in branched chain FAs (pink). Location color bar shows the location of each protein in the cytosol (magenta), mitochondria (yellow) and peroxisomes (red). (**C**) Heat map showing expression of genes involved in fatty acid synthesis. Class bar shows genes for Acetyl-CoA transferases (blue), palmitoyl synthesis (green), elongation (orange), thioesterases (gray) and desaturases (pink). Location bar shows their cellular localization in cytosol (cyan), mitochondria (yellow), peroxisome (black), cellular side of endoplasmic reticulum (red) and endoplasmic reticulum lumen (gray). The color bar on the right represents the log-fold changes; red shows genes upregulated in tumors and blue shows genes downregulated in tumors as compared to normal samples. (**D**) Radar plot showing the total number of genes significantly (LFC > 0.5, FDR < 0.05) upregulated (red) or downregulated (blue) in tumors compared to normal tissue for each cohort. The full names of genes are found in Supplementary Table S3.

β-oxidation is the process by which FAs are broken down into acetyl-CoA to be further oxidized into CO_2_ in the TCA cycle. β-oxidation is separated between the cytosol and the mitochondria (Fig. 3A). In the cytosol, FAs of various lengths are activated to their acyl-CoA forms by certain members of Acyl-CoA Synthetases (ACS) by using ATP^52^. In the mitochondrial matrix, systematic trimming of two-carbon units from the FA chains leads to complete oxidation to CO_2_ via the TCA cycle with the generation of reducing intermediates. There are a total of 26 ACSs in the human genome classified into four subfamilies and named according to the length of FA substrates they catalyze^53^. Their precise role and cellular localization is still under debate as the activated acyl-CoA byproducts can be used either for FAS or FAO^51^. For ease of understanding, we have evaluated the pan-cancer expression of all long (ACSL) and very long (FATPs) ACSs with oxidative enzymes.

ACSL1 is the best characterized enzyme among ACSs, and studies have implicated it in the channeling of acyl-CoA as well as triacylglycerols (TAG) into β-oxidation, but these functions are not universal and may vary in a tissue-dependent manner^52^. Other studies have shown ACSL1 to have a higher impact on β-oxidation in highly oxidative tissues such as cardiac muscle and white/brown adipose tissues. The expression of ACSL1 was decreased by more than 1 LFC in CHOL, LUSC, BRCA, HNSC and LIHC (Fig. 3B). In LIHC, low expression of ACSL1 also showed a significant association with poor prognosis (HR 1.6, p = 0.008). ACSL4, another member of the family, was strongly upregulated in LIHC (> 2 LFC). This protein is implicated in the acylation of the ω-6 fatty acid arachidonic acid and its suppression in HCC cell lines was shown to decrease their proliferation^54^. ACSL4 has also been strongly implicated in susceptibility of cells to ferroptosis by enhancing the ω-6 fatty acid content in membranes^55^. ACSL6 has been implicated in phospholipid synthesis^56^, and it showed strong upregulation (> 1 LFC) in 5 tumor types and strong downregulation in 9 others (Fig. 3B). However, the clinical significance of this finding is unclear and needs to be assessed in future studies. Very little is known about the ACS bubblegum family of genes (ACSBG1/2); both members of the family showed strong up- and downregulation in different tumors, but in absolute terms their read counts were generally low^52^.

In contrast to ACSLs, FATP1-6 (SLC27A1-6) are mostly involved in cellular uptake and activation of very long FAs, with the exception of FATP5 which activates bile acids entering the liver^57^. The expression of FATP5 was suppressed in LIHC, which was associated with poor prognosis (HR 1.5, p = 0.02). The most dysregulated FA transporter among tumors was FATP2, showing significant up- and downregulation in many tumor types (Fig. 3B). Low FATP2 expression was associated with significantly poor prognosis in KIRC (HR 2.8, p = 4e−10), while low expression of FATP1 was associated with significantly poor prognosis in BRCA (HR 1.5, p = 0.03) and LUAD (HR 1.5, p = 0.001). Finally, FATP6 was observed to be the most downregulated gene of all, however the read counts were very low and its real significance in the tumor specimens cannot be determined, especially given the very little information available about its function.

The next step in β-oxidation is the transfer of acyl-CoAs from the cytosol into the mitochondrial matrix by the carnitine palmitoyltransferase (CP) system on the mitochondrial membrane. This step is deemed as the rate-limiting step of FAO as any acyl-CoA entering the mitochondria is funneled into the TCA cycle to be completely oxidized. CP consists of four main components: (1) CPT1 (CPT1A/B) on the outer mitochondrial membrane which conjugates acyl-CoAs to carnitine forming acylcarnitine; (2) CACT, which transfers acylcarnitine to the mitochondrial matrix from the cytosol and carnitine in the opposite direction; (3) CPT2, which exchanges carnitine for CoA, re-forming acyl-CoA inside the matrix; and (4) CRAT (known as CROT in peroxisomes), which re-conjugates carnitine to an acetyl-CoA moiety forming acetyl-carnitine to be transported back to cytosol and completing the carnitine cycle^58–60^. Our data show that CPT1A was strongly downregulated (> 1 LFC) in COAD and KIRP and did not show any significant changes in other tumors. On the other hand, CPT1B was strongly upregulated in 8 tumors. Although it would be tempting to speculate that this enhanced expression leads to increased β-oxidation, the expression of other enzymes involved in β-oxidation did not support this view with only modest changes in expression. Peroxisomes use ABCD channels for the transfer of lipids. We observed increased expression of ABCD1 and decreased expression of ABCD2 in different tumor types (Fig. 3B).

A marked decrease in the expression of Acyl-CoA dehydrogenase (ACAD) enzymes for short (ACADS), medium (ACADM), long (ACADL) and very long fatty acids (ACADVL) was observed in almost all tumor types except KICH. These enzymes catalyze the first reaction of FAO in the mitochondrial matrix by transferring electrons to FAD^+^, which in turn transfers them to the electron transport chain (ETC) for ATP production. On the other hand, ACADs involved in oxidation of amino acid acyl chains (ACAD2, ACAD5 and ACAD8), with few exceptions were generally either downregulated or showed no significant changes. A similar picture emerged for peroxisomal FAO which shortens very long-chained FAs and then transfers them to the mitochondria for final degradation. The pathway is similar to β-oxidation, except that the first and second steps are catalyzed by Acyl-CoA Oxidases (ACOXs) and the peroxisomal bifunctional enzyme enoyl-CoA hydratase and 3-hydroxyacyl-CoA dehydrogenase (EHHADH), respectively. These enzymes were either strongly suppressed (e.g. ACOX2) or showed no significant change. An exception was ACOXL, which showed a mixed pattern of increased and decreased expression in different tumors. Interestingly, ACOXL, a protein of unknown function, was reported as a potential biomarker of benign prostate cancer^61^ (Fig. 3B). However, its absolute expression was very low across all tumors, so the drastic changes we observed in its expression may be an artifact of low read counts. The expression of other enzymes involved in pathway was either slightly decreased or remained unchanged.

Ketone bodies are generated in the liver and serve as an important energy source for the brain, as well as skeletal and cardiac muscles, especially during long periods of exercise or fasting^62^ (Fig. 3A). The rate-limiting enzyme for ketogenesis is HMGCS2, which synthesizes HMG-CoA; the latter is catabolized into acetyl-CoA and acetoactetate by HMGCL, and finally to β-hydroxybutyrate (βOHB) and acetate by BDH1. All genes involved in ketone body synthesis were significantly downregulated in LIHC with both HMGCS2 and BDH1 associated with poor prognosis (HR 2.1, p = 1e−05 and HR 1.8, p = 0.003, respectively). A similar pattern was observed in KIRP (HMGCL:HR 3, p = 0.001 and BDH1: HR 2.4, p = 0.006, respectively) and KIRC (HMGCS2: HR 1.7, p = 7e−04). In addition, contrary to the findings of a study conducted by Chen et al.^63^, where higher expression of HMGCS2 in colon cancer was associated with poor prognosis, in the current study we observed that HMGCS2 was significantly downregulated in COAD, which was associated with poor prognosis (HR 1.6, p = 0.03) (Supplementary Fig. S4A). Supporting this, we observed reduced expression of other enzymes involved in ketone body metabolism, especially HMGCL, which was also associated with poor prognosis in COAD (HR 1.6, p = 0.02). βOHB is synthesized in the hepatic tissue, transported to the bloodstream via SLC16A6 and internalized by cells of extrahepatic tissues, particularly the brain, via the transporter SLC16A1/7. The compound is then either oxidized and channeled into the TCA in the mitochondria, or used for de novo FAS in the cytoplasm. To our knowledge, the current study provides the most comprehensive analysis of expression of genes involved in FAO and ketone body synthesis, and our results lend evidence to clinical studies showing that ketogenic diets improve the outcome of cancer chemotherapy as well as patients’ overall well-being^64^. The overall reduction in FAO and ketone body utilization in many tumor types observed in the current study could be the result of hypoxia and thereby a lack of oxygen for OXPHOS, mitochondrial damage in tumors, or because the tumor cells are unable to conduct catabolism and anabolism simultaneously (FAO and FAS). These need to be better evaluated in future in vivo studies.

#### Fatty acid biosynthesis

FAS occurs in the cytoplasm where citrate produced in the mitochondrial matrix from the TCA cycle is transferred and catabolized into oxaloacetate (OAA) and acetyl-CoA by ATP-Acyl Lyase (ACLY) (Fig. 3A). The cytosolic acetyl-CoA generated is then condensed with CO_2_ to form malonyl-CoA by acetyl-CoA carboxylase 1 (ACACA). The expression of ACLY was significantly increased in 15/20 tumors and only decreased significantly in KICH. The expression of ACACA was also observed to increase in some but not in all tumors. The malonyl-CoA generated has two functions: it serves as the basic unit for fatty acid synthase (FASN) to increase the nascent FA chain by two carbons per cycle; it also inhibits CPT1, thereby preventing a futile cycle of simultaneous FAS and FAO. After the chain length reaches 16 carbon units (palmitate), it is released from FASN to be further processed by elongase and desaturase enzymes into more complex lipids^65^ (Fig. 3A). ACACB is the second isoform of the carboxylase and studies have shown it to be located on the mitochondrial membrane. It has been suggested that the malonyl-CoA synthesized by this enzyme is used exclusively for inhibiting CPT1 and thus inhibit β-oxidation^66^. The same group reported in a later study that mice with mutated (non-functional) ACACB showed increased β-oxidation and did not accumulate fat in their livers^67^. This is in contrast to our observations which suggest that β-oxidation is suppressed in most tumors despite an overall downregulation in the expression of ACACB (except for KICH), potentially refuting the idea that lower ACACB leads to increased β-oxidation. This discrepancy may have arisen because the levels of malonyl-CoA were shown to remain unchanged in the liver of mutant ACACB mice, and were reduced only in the heart and muscles, two organs that are already known to be highly oxidative and relying on β-oxidation. The expression pattern of enzymes involved in the elongation and desaturation of FA showed remarkable differences among tumors, which most likely contributes to major differences in their lipid profiles with potentially different outcomes in cellular signaling, membrane dynamics and cellular behavior (Fig. 3C). Members of short (ACSS) and medium (ACSM) subfamilies of ACS are involved in many functions in the mitochondria such as ketone body and propionate metabolism. ACSs showed drastic decreases in expression with the exception of ACSM4 (Fig. 3C), which in absolute terms had very low expression in all the tumors studied here, and ACSS1 which is involved in propionate metabolism^68^.

Another important class of enzymes involved in FAS and FAO is the acyl-CoA thioesterases (ACOT) family which consists of 12 members and carries out the opposite function of ACSs, namely removing CoA groups from Acyl-CoAs to produce FFAs^69^. Based on their molecular weight, these enzymes are divided into two groups, Type I (ACOT1/2/4/6) and Type II (the rest), and despite being characterized a long time ago, their precise roles are still unclear. However, it is generally assumed that Type I enzymes are mostly involved in FAO, while Type II enzymes play roles in regulating the channeling of fatty acids between FAS and FAO^70^. Our data show that ACOT1/2 were downregulated in most of the tumors except KICH, while ACOT4/6 showed more nuanced patterns. On the other hand, ACOT7 and ACOT11 were significantly upregulated in almost half of the tumors (Fig. 3C). Recently, there have been a number of studies emphasizing the importance of ACOTs as tumor biomarkers. Xu et al.^71^, showed that ACOT8 and ACOT11 were significantly correlated with patient survival in KIRC, while Hung et al.^72^, showed the same for ACOT11 and ACOT13 in LUAD. Since their precise role is not known, further studies in the future will be required to shed more light on the significance of their dysregulation in tumors.

The expression of enzymes involved in mitochondrial fatty acid biosynthesis (FAS-II) was also evaluated in different tumors because the end-product of this pathway is necessary for the production of lipoic acid, an essential component of the enzymes pyruvate dehydrogenase (PDH) and 2-oxoglutarate dehydrogenase (OGDH)^73^ (Supplementary Fig. S4B). Studies have shown that suppression or overexpression of some of the FAS-II genes has a large impact on mitochondrial function, especially ATP production^74,75^. Our data suggest modest changes in the expression of most enzymes of this pathway, except ACSM1 which showed increased expression (> 1 LFC) in three tumor types (KIRP, LIHC & PRAD) and showed decreased expression in most of the others. ACSM1 has been implicated in the activation of lipoic acid in the mitochondria, but whether the changes observed here have any impact on mitochondrial function cannot be determined solely from transcriptomic data. Additionally, the expression of genes involved in cardiolipin synthesis or modification remained mostly unchanged except GPAT2 and THEM5, which were significantly overexpressed in eight tumors (Supplementary Fig. S4B).

Overall, our analyses suggest that most of the tumors showed downregulated fatty acid metabolism, especially the oxidative branch (Fig. 3D). The only tumor type having significantly more upregulated genes was UCEC while the opposite was true for 12 tumors with CHOL showing the highest number of downregulated genes. PAAD and ESCA showed the same pattern with carbohydrate metabolism, most likely due to their high heterogeneity as mentioned before. We also evaluated the possibility of some of the enzymes identified in the current study for their use as novel biomarkers in the future. Among the most significant ones are the enzymes involved in ketone body metabolism for LIHC, KIRP and KIRC.

### Amino acid metabolism

As building blocks of proteins and the source of amino groups in the cell, amino acids (AAs) are essential for cellular growth and survival. Among the 20 AAs used for protein synthesis, eukaryotic cells cannot synthesize nine; these are therefore essential amino acids (EAAs) that need to be obtained from the diet. Seven amino acids are synthesized as byproducts of the EAAs, and only 4 can be synthesized by simple transamination reactions, so they are known as non-essential amino acids (NEAA)^76^.

**Figure 4:**
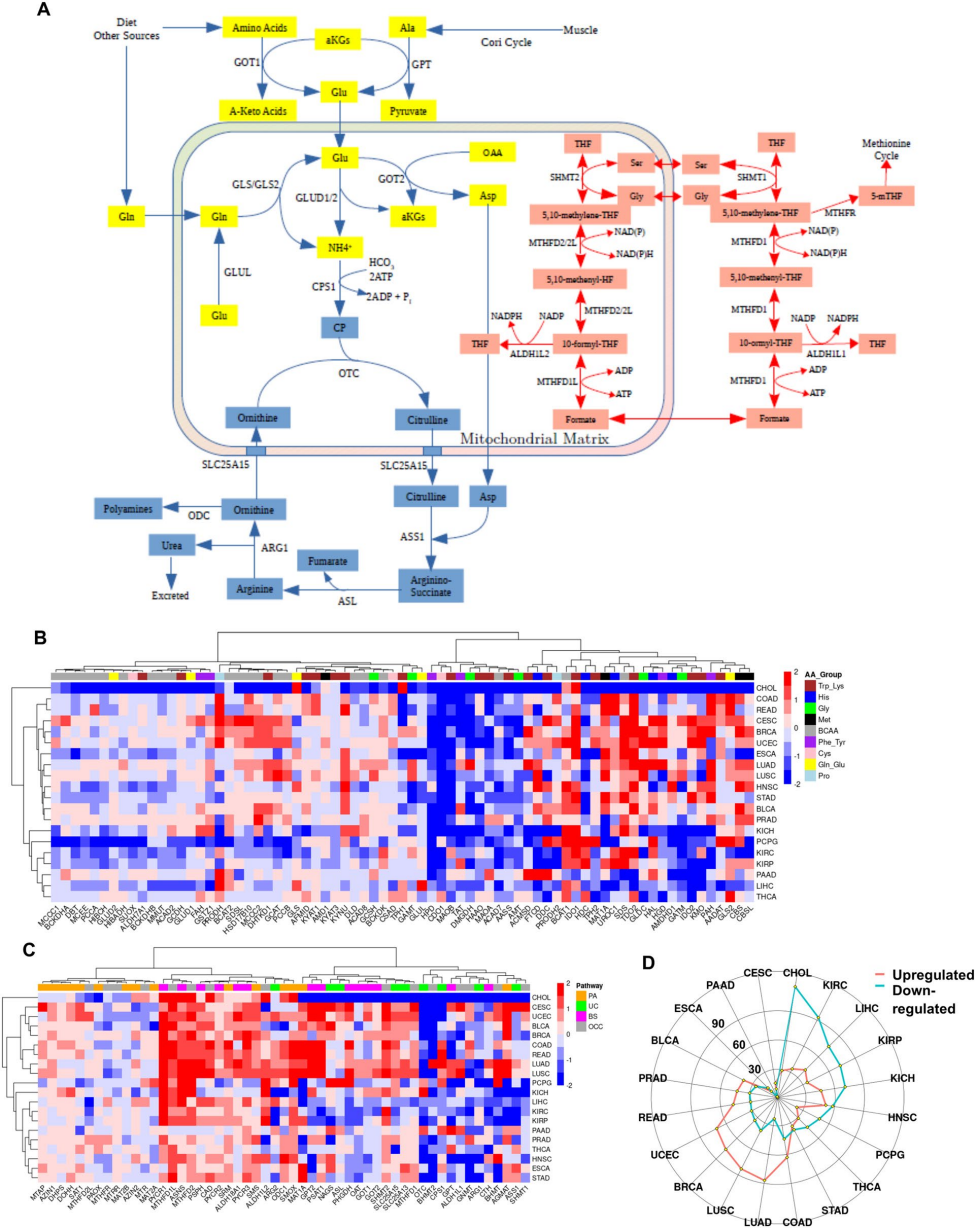
Alterations in enzymes of amino acid metabolism between cancer and matched normal samples. (**A**) A simplified schematic representation of amino acid metabolism, particularly glutamine and glutamic acid (yellow), urea cycle (blue) and one carbon cycle (red). (**B**) Heat map showing the expression of genes involved in amino acid oxidation. Pathways were grouped according to their cross-talk. Color bar shows enzymes involved in tryptophan-lysine (Trp-Lys), histidine (His), glycine (Gly), methionine (Met), branched-chain amino acids (BCAA), phenylalanine-tyrosine (Phe-Tyr), cysteine (Cys), glutamine and glutamate (Gln-Glu) and proline (Pro). (**C**) Heat map showing the expression of genes involved in amino acid biosynthetic pathways. Color bar represents genes involved in Polyamine pathway (PA, orange), Urea Cycle (UC, green), Biosynthesis and Transamination reactions (BS, pink), and one carbon cycle (OCC, gray). The color bar on the right represents the log-fold changes; red shows genes upregulated in tumors and blue shows genes downregulated in tumors as compared to normal samples. (**D**) Radar plot showing the total number of genes significantly (LFC > 0.5, FDR < 0.05) upregulated (red) or downregulated (blue) in tumors compared to normal tissue for each cohort. The full list of genes is found in Supplementary Table S5.

Normal as well as transformed cells therefore depend heavily on amino acids obtained from the diet. Studies have shown that the availability of free amino acids (FAAs) supports anabolism and inhibits catabolism, increases protein synthesis, mRNA abundance and its stability via the mTOR pathway, while lower concentrations of FAAs have the opposite effect^77,78^. AAs are also essential for the mitigation of oxidative stress via the formation of glutathione from three AAs (Cys, Gly and Glu), in addition to their role in nucleotide biosynthesis. Glutamine (Gln) comprises the largest pool of FAAs in the human body reaching up to ~ 500 μM in the circulation^79^. Until recently, glutamine [together with alanine (Ala)] was thought to act solely as an amino group carrier from other organs to the liver where excess amino groups were removed in the urea cycle (UC). However, once it was shown to act as major source of energy for rapidly-dividing cells, Gln metabolism attracted a lot of attention in cancer research^80^. AA metabolism is very complex and is highly interconnected with many other cellular processes. To better evaluate their role in the bioenergetics of cancer, we have divided the amino acid related pathways into cellular transport, oxidation and biosynthesis; the latter including the polyamine (PA) pathway, UC and folate, otherwise known as one-carbon cycle (OCC) (Fig. 4A).

#### Amino acid transport

There are more than 60 amino acid transporters (AATs) in eukaryotic cells belonging to 11 solute carrier (SLC) families^81^. Since AA concentrations are higher inside the cell compared to the extracellular environment, their influx has to be coupled to the transport of ions such as K^+^, Na^+^, H^+^, and Cl^+^, or to the efflux of another amino acid, generally Gln or Glu^82^. In addition, mutations of certain AATs have been implicated in number of inborn AA metabolic disorders such as cystinuria, cystiniosis and iminoglycinuria^83^. The expression of AATs varies drastically between different organs, making data interpretation very challenging^84^. Nonetheless, it is obvious from Supplementary Fig. S5 that a great majority of AATs are upregulated in most tumors. The heat map shows two major clusters for AAT expression; a large cluster showing transporters upregulated in tumors, and another one showing the downregulated genes which consist mostly of neuronal transporters that have very low expression in extra-neuronal organs. The precise role of many AATs is not known, but some of them, especially Gln transporters of the SLC1/6/7/38 families are known to play major roles in tumorigenesis. One such example is SLC1A5 which transports Gln into the cell to be later transported out in exchange for EAAs via SLC7A5/SLC3A2. Nicklin et al.^85^, reported that this system was essential for the influx of EAAs, in particular Leu into the cells. AA influx can activate the mTOR signaling pathway, leading to cell proliferation; therefore, inhibition of AA transporters could lead to the deactivation of the mTOR pathway, inhibiting cell proliferation and initiating autophagy. Two additional studies showed that Gln and Ser transporters SLC1A5 and SLC1A4 were essential for progression of PRAD and the knockdown of SLC1A5 significantly decreased tumor progression in vivo^86,87^. The influx of Cys, a component of glutathione, is coupled to the efflux of Glu and its two known transporters are SLC7A11 and SLC3A2. It has been shown recently that SLC7A11 (but not SLC3A2) was essential for the survival of *TP53* mutant cells in vitro and in vivo, and its blockage caused cell death by significantly increasing oxidative stress^88^. Our data show that SLC7A11 was strongly upregulated in all tumors except THCA (Supplementary Fig. S5), indicating that mitigation of oxidative stress was a common theme in most cancers, while the expression of SLC3A2 showed more variation. Survival analysis showed that high expression of SLC7A11 was associated with poor prognosis in BRCA (HR 1.6, p = 0.04), KIRC (HR 1.4, p = 0.02), KIRP (HR 2.8, p = 0.003), and LIHC (HR 1.9, p = 5e−04), while the high expression of SLC3A2 was associated with poor prognosis in BLCA (HR 1.9, p = 0.003) and LUAD (HR 1.6, p = 0.03). Surprisingly, only one of the Gly transporters (SLC6A9) was significantly upregulated in four tumors, BRCA, CESC, CHOL and LIHC. These results may indicate that Gly, a component of glutathione, may be endogenously generated from Ser in the OCC. Overall, our results suggest that high expression of AATs in most tumors enables AA influx, leading to enhanced protein synthesis and protecting tumors from lethal levels of oxidative stress in hypoxic environments.

#### Amino acid oxidation

Amino acid oxidation (AAO) is highly complex, with some pathways having as many as 20 steps, but the end products are all channeled into the TCA to be oxidized and produce energy^76^. For clarity, we have grouped AAs according to their common oxidation pathways. As seen in Fig. 4B, almost all enzymes involved in AAO were downregulated in about half of the tumors. The remaining cancer types showed upregulation of enzymes involved in the oxidation of Met, Gly, His and Pro and the initial steps of Trp oxidation. Of note, upregulation of GLS/GLS2 in many of the tumors suggests enhanced conversion of Gln to Glu, which can then presumably be utilized for the production of the endogenous antioxidant glutathione (GSH) as well as converted to α-Ketoglutarate, which can enter the TCA cycle^89^. The role of Pro catabolism is controversial; some studies have shown Pro oxidation to be involved in the mitigation of oxidative stress, while others have shown that increased expression of proline dehydrogenase (PRODH) was involved in apoptosis and reduced DNA synthesis^90^. In the current study, we observed that PRODH was significantly dysregulated in 14 tumors (7 upregulated and 7 downregulated) (Fig. 4B). PRODH2 was expressed only in LIHC, KIRP and KIRC where its expression in tumors was lower compared to normal tissues. The absolute read counts in the other tumors were very low. Considering the expression of genes involved in both the transport and oxidation of AA, the data suggest that amino acids are most likely not used for energy generation in tumors; rather, AAs appear to be conserved and utilized for mostly protein synthesis or to mitigate oxidative stress.

#### Amino acid synthesis, PA, OCC and UC

Ser is a conditionally non-essential amino acid of major importance for normal cell function because it serves as precursor for the synthesis of Cys and Gly, is incorporated in phospholipids, and donates carbon units to OCC, methionine cycle and de novo nucleotide synthesis (Fig. 4A)^91^. Ser can be synthesized from 3-phosphoglycerate in glycolysis by the rate-limiting enzyme PHGDH or in OCC by SHMT1/2 when ample amounts of Gly are available. Our data show that enzymes involved in Ser biosynthesis through glycolysis (PHGDH, PSAT1, PSPH) were significantly upregulated in CESC, LUAD, LUSC, PCPG, READ, COAD and UCEC. In addition, PHGDH was associated with poor prognosis in BLCA (HR 1.5, p = 0.005), COAD (HR 1.5, p = 0.04) and UCEC (HR 2, p = 0.002). Corroborating this, Maddocks et al.^92^, showed that colon cancer cells rely heavily on Ser available in cell culture, and Ser deprivation slowed down proliferation and increased de novo Ser synthesis in a p53 dependent manner. On the other hand, Ser biosynthesis was downregulated in CHOL, KIRC, KIRP and LIHC, indicating that these tumors may rely on transport of Ser into the cell. Of note, Ser biosynthesis was recently shown to activate mitogenic signaling via mTORC1 in lung metastasis of breast cancer but not in the primary breast tumor. Expression of PHGDH in the lung metastatic tissue was necessary for the sensitivity of mTORC1 to rapamycin^93^⁠. Cys is conditionally essential because it can be synthesized from Homocysteine and Ser by cystathionine gamma-lyase (CTH). We observed that CTH was either downregulated or showed no significant change in expression in most tumor types except LUAD (Fig. 4C). This is a strong indication that tumors mostly import Cys, supporting our observation of enhanced expression of the Cys transporters SLC7A11 and SLC3A2 in tumors compared to normal tissues (Supplementary Fig. S5). Asparagine synthetase (ASNS), which synthesizes Asn from Asp and Gln, was upregulated in almost all tumors (Fig. 4C). This enzyme has attracted attention especially because of the effectiveness of Asparaginase therapy in treating acute lymphoblastic leukemia (ALL), acute myeloid leukemia (AML), and non-Hodgkin's lymphoma^94^. Asparaginase is obtained from bacteria and depletes Asn pools in the tumor, leading to cell death. Further studies have shown that Asn export from the cell is coupled with the influx of His, Arg and Ser, which then activate mTOR pathway in a similar fashion to Leu^95^. Clinical data from the cohorts in the current study showed that higher expression of ASNS was associated with poor prognosis in HNSC (HR 1.4, p = 0.01), KIRC (HR 2.7, p = 7e−10) and LIHC (HR 1.6, p = 0.01).

Enzymes for the biosynthesis of Pro (ALDH18A1/PYCR1/2/3) were also upregulated in almost all tumors (Fig. 4C). Despite the controversy regarding the significance of Pro^90^, this AA appears to be essential for tumor progression. Survival analysis showed that increased expression of ALDH18A1 and PYCR1/2 was associated with poor prognosis in KIRP (ALDH18A1: HR 2.2, p = 0.01; PYCR1: HR 3.0, p = 0.001; PYCR2: HR 2.0, p = 0.03), KIRC (ALDH18A1: HR 1.9, p = 4e−05; PYCR1: HR 2.3, p = 5e−07; PYCR2: HR 1.9, p = 3e−05) and LIHC (PYCR1: HR 1.8, p = 0.01, PYCR2: HR 1.5, p = 0.04), indicating that Pro biosynthesis may be an important factor in survival and development of these three tumors. Alanine (GPT/GPT2) and Aspartate (GOT1/GOT2) transaminases are involved in transferring amino groups from Ala and Asp respectively, to pyruvate and αKG (Fig. 4A). GPT is especially important for the conversion of pyruvate produced in muscles during exercise to Ala, which is then converted back to pyruvate and glucose in the liver via the Cori Cycle. Our data show that the cytoplasmic form of the transaminase (GPT) was downregulated in most tumors; additionally, this downregulation was associated with poor prognosis in LIHC (HR 1.7, p = 0.003) where this enzyme is of fundamental importance, especially for gluconeogenesis. However, GPT was upregulated in CESC, LUAD, LUSC and PAAD, which may be associated with channeling of pyruvate into the mitochondria. This is an alternative entry way of pyruvate into the mitochondrial matrix and becomes essential when pyruvate transporters (MPC1/2) are downregulated or knocked down^96,97^. The mitochondrial isoform (GPT2), which interconverts αKG into Glu was upregulated in a number of tumors. On the other hand, GOT1/2 which are involved in the Malate-Aspartate shuttle showed mild, mostly non-significant changes between tumors and their normal adjacent tissues. Based on transcriptome data, this suggests that the transfer of electrons generated from glycolysis into the mitochondria for OXPHOS was not prioritized in tumor cells; however, further regulation at the protein or metabolome level are likely to occur.

The urea cycle (UC) mostly takes place in the liver where ammonia is collected from other parts of the body in the form of Gln and Ala to be converted to urea and excreted (Fig. 4A). Therefore, normal functioning of this cycle in the liver is essential to avoid ammonia toxicity. In extra-hepatic tissues, UC serves as the main source of Arg, citrulline and ornithine^98^. Our data show that the two rate-limiting enzymes of the UC, namely carbamoyl phosphate synthetase 1 (CPS1) and ornithine transcarbamoylase (OTC) were downregulated in LIHC (Fig. 4C), and not surprisingly, this downregulation was associated with poor prognosis (OTC HR 1.5, p = 0.03, CPS1; HR 1.8, p = 0.001). The enzyme OTC, which condenses carbamoyl phosphate (CP) and ornithine to form citrulline, and ARG1 which converts Arg to ornithine were downregulated in most tumors, while argininosuccinate lyase (ASL), which synthesizes Arg and fumarate from arginosuccinate showed variation in expression between different tumor types (Fig. 4C). Ammonia is also captured in the cytosol by carbamoyl-phosphate synthetase 2, aspartate transcarbamylase, and dihydroorotase (CAD), a cytosolic multifunctional enzyme that uses CP for pyrimidine synthesis, the latter being essential for the generation of nucleotides^99^. Our data show that CAD was significantly upregulated in the majority of tumors and this upregulation was associated with poor prognosis, especially in LIHC (HR 1.9, p = 5e−04). These observations are in line with recent findings that tumors do not discard ammonia; rather it is utilized for nucleotide, polyamine and amino acid biosynthesis^98^.

Polyamines (PA) are a group of highly charged molecules synthesized by the multistep polyamine pathway (PAA). PAs are involved in multiple cellular processes such as cell signaling, chromatin remodeling and enhancement of protein translation, as well as tumorigenesis. The most important step in PAA is the formation of putrescine from ornithine in presence of the enzyme ornithine decarboxylase 1 (ODC1), and subsequently the formation of spermine and spermidine. These three compounds comprise the majority of PAs found in the cell^100,101^. Our data show that ODC1 was significantly downregulated in BLCA, KICH and PAAD and significantly upregulated in 10 other tumors (Fig. 4C). A similar pattern was observed for SMS, SMOX and SRM, all of which were associated with poor prognosis in LIHC, while the others did not show any highly significant difference (SMS; HR 1.9, p = 7e−04; SMOX: HR 1.7, p = 0.003; SRM: HR 1.7, p = 0.03). This expression profile may explain why the UC is generally downregulated in LIHC; the liver is a repository of ammonia and instead of excreting it via UC, the tumor utilizes it for polyamine and nucleotide biosynthesis in order to enhance its survival and progression.

Overall, our study has shown a significant overexpression of genes involved in AA transportation through the plasma membrane and activation of pathways utilizing glutamine which may serve as an amino group donor for nucleotide biosynthesis, for transamination reactions, or enter TCA either to be used for energy generation or to be funneled to FAS. In addition, UC was shown to be suppressed while PA was activated, especially in LIHC, a state associated with poor overall patient survival. These pathways and their significance, especially in liver cancer will need more elaborate studies in the future because urea metabolism is one of the major functions of this organ. The deregulation of genes (either up or down regulation) was evenly balanced across tumor types (Fig. 4D).

### TCA, anaplerosis and ETC

#### TCA and anaplerosis

**Figure 5:**
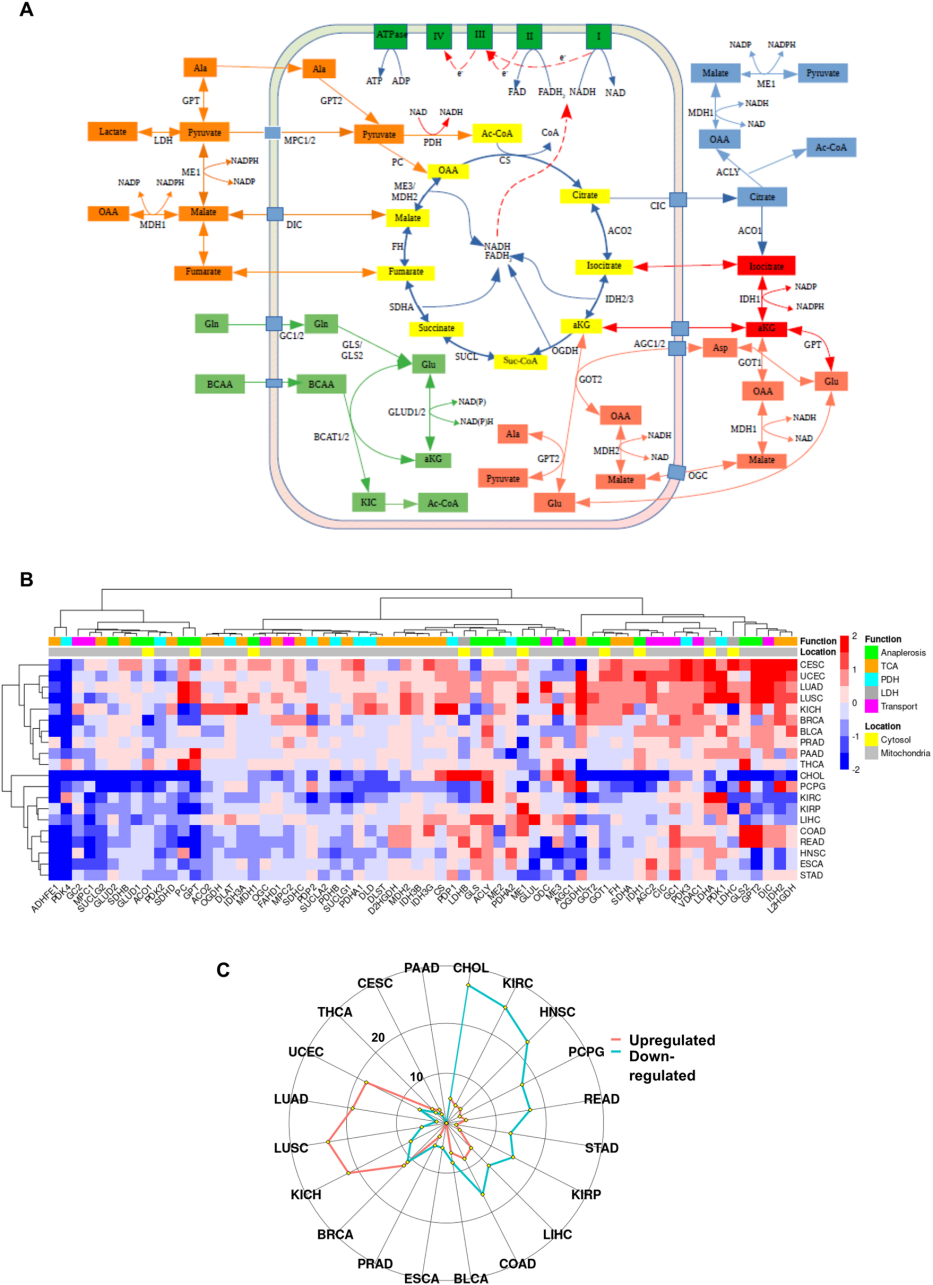
Alterations in enzymes of TCA cycle and anaplerosis between cancer and matched normal samples. (**A**) A simplified schematic representation of tricarboxylic acid (TCA) cycle and anaplerotic pathways. TCA cycle reactions are shown in yellow, pyruvate-malate cycle is shown in orange, glutamine and branched-chain amino acid (BCAA) in light green, pyruvate-citrate cycle in blue, isocitrate-alpha-ketoglutarate in red and malate-aspartate shuttle in pink colors. Finally, electron transport chain complexes and ATP synthase are shown in dark green. A number of important transporters are represented as blue color boxes on the mitochondrial membrane. (**B**) Heat map showing expression of TCA cycle and anaplerosis enzymes. Function bar shows in color code genes involved in anaplerosis (green), TCA (orange), pyruvate dehydrogenase complex members (cyan), lactate dehydrogenases (gray) and certain transporters (pink). Location bar shows their cellular localization, in cytosol or mitochondria. The color bar on the right represents the log-fold changes; red shows genes upregulated in tumors and blue shows genes downregulated in tumors as compared to normal samples. (**C**) Radar plot showing the total number of genes significantly (LFC > 0.5, FDR < 0.05) upregulated (red) or downregulated (blue) in tumors compared to normal tissue for each cohort. The full names of genes are found in Supplementary Table S6.

The tricarboxylic acid (TCA) cycle, besides being the main source of cellular ATP, is also essential for anaplerotic reactions since its intermediates can be inter-converted to amino acids, channeled into FAS and used for transferring reducing electrons by means of NADH/NADPH via various mitochondrial shuttles (Fig. 5A). Overall, our data support a highly complex, tumor specific regulation of these pathways (Fig. 5B). Four tumors (CESC, UCEC, LUAD and LUSC) and to a certain extent four others (KICH, BRCA, BLCA and PRAD), exhibited a general tendency of enhanced anaplerosis as reflected by an upregulation of genes related to the TCA pathway, as well as enzymes involved in glutaminolysis and transamination reactions inside and outside the mitochondria (Fig. 5B). In addition, these same tumors showed a general increase in the expression of Gln and Glu transporters. Thus, while the expression of the glycolysis genes was almost universally upregulated in the different tumor types, regulation of the TCA enzymes appear to be more complex with tumor specific regulatory mechanisms (Supplementary Fig. S1).

Importantly, we also observed that pyruvate metabolism was not only regulated by the decreased expression of pyruvate dehydrogenase (PDH) and upregulation of pyruvate kinases (PDKs), but also by a downregulation of its transporters, especially MPC1 which showed at least 1 LFC downregulation in 9 tumors, while MPC2 was mildly upregulated (< 1 LFC) in only four of them, with the rest having non significant changes (Fig. 5B). Studies have shown that depletion of MPC1/2 in myocytes grown in normoxic condition causes metabolic reprogramming, forcing the cells to rely more on oxidative glutaminolysis to replenish the TCA intermediates^102^. On the other hand, under hypoxia-induced suppression of PDH, cells rely more on IDH1-driven reductive carboxylation to generate citrate for FAS^103^. PDH also did not show any marked decrease, but PDK1 and PDK3 were upregulated in some of the tumors, pointing at additional mechanisms for suppressing the normal functions of PDH (Fig. 5B).

The rest of the enzymes involved in TCA did not show any notable change, except for IDH2, which may be involved in shifting the TCA cycle in the reverse direction by using NADPH to generate citrate for FAS. The expression of this enzyme was significantly increased in seven tumors and decreased in five others (Fig. 5B). Two enzymes that have attracted a lot of attention from cancer researchers in recent years are IDH1 and ME1. Both enzymes are important for anaplerosis and especially for the reduction of NADP into NADPH. ME1 was significantly upregulated in KIRP, COAD, LUSC and LIHC; in LIHC it was associated with poor prognosis (HR 1.7, p = 0.003). The enzyme was downregulated in PCPG, BRCA, STAD and PRAD. IDH1 was strongly upregulated in UCEC and mildly in some other tumors. The same cluster of tumors also showed increased expression of Glu, Gln, Malate, Citrate, Asp and α-KG transporters, which may indicate that these tumors relied heavily on glutamine anaplerosis to generate the necessary citrate for FAS. These tumors, together with KIRC, also showed higher expression of lactate dehydrogenases A and B (LDHA/B). One study has shown that high expression of LDHA was associated with poor prognosis in LUAD but not LUSC^104^. Overall, our data show that changes in expression of genes involved in TCA and anaplerosis were not as drastic as in other pathways, however almost half of the tumors had significantly more downregulated genes as compared to normal tissues. On the other hand, KICH, LUSC, LUAD and UCEC showed the opposite trend (Fig. 5C).

#### Electron transport chain

Electron Transport Chain (ETC) complexes are composed of multiple protein subunits, the majority of which are still not well characterized or have no direct role in electron transfer; therefore, based on the available literature we evaluated the expression of only the core subunits encoded by mitochondrial and nuclear genomes^105,106^. ETC is not only important for ATP synthesis, but also serves as a major source of ROS, which has been implicated in many cellular processes such as cellular differentiation, genomic instability and increased cellular survival or death, depending on the amount of ROS and the cellular context^107^. These observations are in line with the “ROS Rheostat Model”, according to which, depending on the amount generated, ROS may act as a tumor inducer or suppressor^108^. Our data suggest that mitochondrial-encoded genes of the ETC and ATP synthase were generally downregulated in almost half of the cancers evaluated and upregulated in the remaining half (Supplementary Fig. S6A). A cluster of seven tumors showed a tendency for overexpression of certain components of the ETC, which may be interpreted as more active TCA since the same cancers had higher expression of TCA and anaplerotic enzymes (Fig. 5B). However, for the same tumors we also observed an increase in the expression of uncoupling enzymes, especially UCP2, which has been implicated in protecting tumors from excessive ROS^109^ and was recently shown to enhance tumorigenesis by inducing metabolic reprogramming towards anabolism^110^. Other UCPs have been associated with various cellular phenomena, mostly thermoregulation (UCP1) and neurodegeneration (UCP4 and UCP5)^111^, but the real significance of these enzymes in tumorigenesis remains to be established.

ETFA, ETFB and ETFDH are the proteins involved in the transfer of electrons from amino acids and β-oxidation to ETC complex II. ETFDH was downregulated in most of the tumors, which is supported by a decrease in enzymes of β-oxidation (Supplementary Fig. S6A). The glycerol 3-phosphate dehydrogenase (GPD) system, known also as glycerophosphate (GP) shuttle, plays a major role in cellular bioenergetics, with the mitochondrial isoform (GPD2) being an integral part of the ETC. The shuttle’s main function is to oxidize cytosolic NADH by GPD1 and transfer the electrons to FAD of GPD2 for ATP production in mitochondria^112^. Another member of this shuttle is GPD1L, which is thought to have similar roles with GPD1. GPD1 and GPD1L were downregulated in most tumor types; low GPD1L was associated with poor prognosis in COAD (HR 1.6, p = 0.03), LUAD (HR 1.7, p = 4e−04) and KIRC (HR 1.8, p = 3e−04), while higher expression was associated with poor prognosis in LIHC (HR 1.7, p = 0.005). The mitochondrial isoform did not show significant changes in expression except in CHOL, PAAD and CESC, and studies have shown that increased expression of this enzyme is associated with increased glycolysis and ROS levels in prostate tumor cell lines^113^. Overall, the cohorts can be divided into three major groups based on DGE of ETC genes; those that had a high number of downregulated genes, those that showed the opposite trend, and those that showed not much change (Supplementary Fig. S6B).

## Conclusions

Energy is the basis of life, or as Franklin Harold put it in his seminal work on bioenergetics, “the vital force”^114^. All cells require energy, whether in their dividing state for anabolism, or during senescence in order to maintain their biochemical machinery. Since cancer cells are in a constant state of division, they need to make major metabolic rearrangements to cope with anabolic demands. In our opinion, one of the most accurate definitions of a tumor is that of a parasite whose only purpose is to reproduce and grow at the expense of its host^115^. In order for the parasite to grow, it needs to redirect the flow of intermediary metabolism to gain the most out of it. A major distinguishing feature of tumors is aerobic glycolysis, or Warburg effect, a process that is thought to confer three main benefits to malignant cells: first, biosynthesis, especially via the reducing factor NADPH, which is essential for lipid and nucleotide biosynthesis; second, invasion of adjacent tissue and protection from an immune response by the production of copious amounts of lactate which reduces the pH of the surrounding environment; and third, a survival advantage when oxygen is a limiting factor under hypoxia^29^.

Breast cancer is highly heterogeneous with five different molecular subtypes identified (Basal, Luminal A, Luminal B, HER2 and Normal-like) and are known to be metabolically very heterogeneous as well^116^⁠. A recent multi-omics study conducted on breast cancer samples also corroborated the intertumor metabolic heterogeneity, but could cluster the tumors into two categories: those that were highly glycolytic and associated with poor prognosis and those that preferentially used fatty acid oxidation or glutaminolysis and were associated with better prognosis^117^⁠. We also observed high heterogeneity in expression in breast cancer and matched normal samples. However, genes that regulate flux through glycolysis, PPP or TCA could separate tumors from matched normals (Supplementary Fig. S7). More importantly, we observed that several glycolytic, FAS and PPP genes (ME1, ACLY, G6PD, PFKL, IDH3G, PDK3, HK1, IDH2, MCT4) were upregulated in nearly all BRCA specimens compared to normals, irrespective of their molecular subtype (Supplementary Fig. S7, genes represented within the yellow box).

One overlooked aspect of glycolysis is its role in providing acetyl-CoA for FAS, partially because a major portion of current research assumes that all the pyruvate produced by glycolysis is converted into lactate due to the decrease in expression or activity of PDH, and also because of heavy focus of clinical research on approaches that interfere with DNA replication. However, only a fraction of pyruvate is converted into lactate, while the rest enters mitochondria and TCA. Moreover, there are also other alternative ways for pyruvate to enter mitochondria through anaplerosis. In addition, it is also generally assumed that lactate is a waste byproduct of the Warburg effect to be excreted by the cell, but recent metabolomics studies in mice have shown that tumors are able to metabolize lactate for energy production^118,119^. These recent findings are in line with a model proposed by Costello & Franklin according to which glycolysis is essential for tumors to produce acetyl-CoA which is used for lipogenesis and cholesterologenesis. Pyruvate can be used to produce acetyl-CoA directly, as well as replenish OAA by means of anaplerosis because OAA is depleted when there is a high demand for FAS. Biochemical calculations also show that this is an optimal choice for cancers to produce the required energy in the form of ATP for anabolism and secure the necessary amounts of acetyl-CoA^115^. Very detailed biochemical studies gathered by Pedersen^120^ in the past have shown that tumor cells derive 60–85% of their ATP from mitochondria, which may come from OCC, ETC or substrate-level phosphorylation by succinate dehydrogenase^121^. These studies also showed that the more aggressive the tumor cells, the higher their glycolytic rate and the lower their mitochondrial content as compared to normal cells^120^.

The best way to understand the flow of metabolic pathways is to quantify the metabolites in a particular tissue. However, despite all the advancements in mass spectrometric approaches and software development, isolation, quantification, characterization and identification of metabolites is currently a daunting task^122^. The use of transcriptomics as a surrogate for metabolomics relies on strong correlation between transcript levels of metabolic enzymes and their corresponding metabolites. This was confirmed in a breast cancer cohort where metabolite levels were found to be correlated with RNA-Seq data^123^. Therefore, with some caution, we can claim that the data presented here can be a good representation of the real state of the metabolome in tumors.

In the current study, we have shown that tumors show enhanced uptake of carbohydrate and amino acid by overexpressing certain families and isoforms of their transporters. This, to our knowledge is the first systematic study to analyze the mRNA expression of this important group of proteins and evaluate their relevance in tumor metabolism. Although the genes evaluated in this study were manually curated, potentially opening up possibilities of a bias in selection, we ensured to the best of our knowledge and data available in the literature that the genes represented all relevant pathways. Supporting the extensive data available in the literature, we have shown that glucose is used in glycolysis, while AAs, with the exception of glutamine are not catabolized for energy generation, but are funneled into protein synthesis. Our data also suggest that all tumors showed elevated FAS and decreased FAO, a state that is in agreement with the anabolic nature of cancers. Another important and common feature of tumors is a decrease in the utilization of ketone bodies for the purpose of energy generation. These data support findings from several pre-clinical studies showing better response to cancer chemotherapy of patients who underwent intermittent fasting or were kept in ketogenic diet. Our data also corroborates recent suggestions that cancers can be managed by means of dietary changes, however the data on this area are scattered and there is no consistent dietary regime for cancer patients^124^. In addition, we have determined the correlation of certain key enzymes with patient survival and found several significant associations.

To minimize confounding effects, we have chosen exclusively treatment-naive tumor samples that also had data for matched normal tissues. The last factor was important to mitigate variations coming from different genetic/epigenetic backgrounds, as well as the variations coming from the organs themselves. However, it needs to be emphasized that although the matched normal samples were collected > 2 cm away from the tumor and were histopathologically free of tumor cells^125^⁠, these tissues are known to be different from healthy tissues, expressing a pro-inflammatory gene signature^126^⁠ and contributing to patient survival across cancer types^127^⁠. Transcriptome data from healthy tissues that are collected from a distance greater than 2 mm are not available for the TCGA cohort and the use of expression data from healthy tissues that are independently collected and processed may lead to significant disparities that may make data interpretation difficult.

Although this study is not the first to use pan cancer transcriptomics to understand metabolism, it has its own unique features. First, instead of using ready-available pathways, we used a more targeted approach of the most important and relevant enzymes and transporters. Although systemic approaches are more appealing, they are sometimes less informative because metabolic pathways are highly interconnected, and all the enzymes in the network are given the same weight, missing the more important ones. Second and most important, systemic studies rely heavily on statistics and often do not explain the relevant biochemical pathways in a language that can be understood by most of the biological research community. Our study focuses on a manually curated extensive list of genes that are known to be highly relevant to metabolism in cancers and clearly shows the importance of gene expression changes while providing simplified descriptions of the alterations in metabolic pathways and interconnections between those pathways.

## Methods

### Metabolic pathways

The enzymes and transporters used in this study were gathered through a combination of database querying and manual curation. More specifically, glycolysis/gluconeogenesis and amino acid and nucleotide sugar metabolism (including genes of the hexosamine pathway) related genes were retrieved from the Kyoto Encyclopedia of Genes and Genomes (KEGG) (https://www.genome.jp/kegg/)^128^ while the rest were retrieved from the Molecular Signature Database of the Gene Set Enrichment repository (https://www.gsea-msigdb.org/). They were then manually curated from literature, after which some genes irrelevant for our study were removed and a number of others were added in order to make the pathways more complete and less redundant. In addition, all the enzymes were classified according to their function, and when necessary, their cellular localization. In this way, we created a database of genes involved in bioenergetics representing 5 major pathways: (1) carbohydrate metabolism comprising genes involved in carbohydrate transport, glycolysis, pentose phosphate pathway and hexosamine biosynthesis pathway; (2) fatty acid metabolism, comprising genes involved in fatty acid oxidation, transport through mitochondrial membrane, ketone body oxidation and biosynthesis, cellular fatty acid biosynthesis and modification (FAS-I), and mitochondrial fatty acid biosynthesis (FAS-II); (3) amino acid metabolism comprising of genes taking part in amino acid transportation through the plasma membrane, amino acid oxidation, amino acid biosynthesis, polyamine pathway (PA), urea cycle (UC) and once-carbon cycle (OCC); (4) tricarboxylic acid cycle (TCA) and anaplerotic pathways in mitochondria; and (5) electron transport chain (ETC). All the gene names are available in Supplementary Tables S1–S7.

### Data acquisition

Pre-aligned, raw RNA Sequencing read counts in HTSeq format as well as patient clinical data were downloaded from TCGA database using the *TCGABiolinks* package according to the package’s instructions for transcriptomic data^129,130^. A total of 6043 probes representing genes, various families of short RNAs, pseudogenes and other classes of transcripts were downloaded for each tumor sample and matched with their adjacent normal tissues according to patients’ unique barcodes. Any cohort having less than 3 matched normal samples was discarded from further analysis, and as a result, we were left with 20 cohorts comprising a total of 1386 samples, half of them tumors and the other half matched normal (Fig. 1).

### Data normalization, differential gene expression and visualization

For each cohort, read count normalization and differential gene expression (DGE) analysis for tumor versus normal sample was carried out separately by *DESeq2*^131^ package according to instructions. Briefly, a general linear model (GLM) was built for each tumor using the samples status (tumor versus normal) as variable and all probes having no read counts were filtered out for easier computation. Finally, differential gene expression dataframes for log-fold change differences of tumor-normal were obtained to be used for further analysis. Each gene’s significance was determined by Wald Test’s p-value adjusted for multiple testing by Benjamini and Hochberg procedure false discovery rate (FDR)^131^⁠ < 0.05 and log-fold change (LFC) higher than 0.5. Genes that were upregulated, downregulated or did not show any statistically significant difference in expression according to the conditions mentioned above are listed in Supplementary Tables S9–S13 and visually in the radar plots. LFC values for each gene of interest were extracted from expression matrices and recombined with other necessary information into separate dataframes for heatmap construction. All heatmaps were constructed using *pheatmap* package with euclidean distance and ward.D2 linkage method. Radar plots were generated with *ggplot2* package and the number of genes up- or down-regulated for each cohort were determined according to the criteria mentioned above. Additionally, we validated sample separation (tumor vs normal) for each cohort by PCA using vst-transformed read counts.

### Survival analysis

For survival analysis, certain rate-limiting enzymes or transporters were tested for possible correlation with patients’ survival. The criteria for inclusion in analysis were for the genes to show significant difference in expression between tumor and normal as well as correlation being in the same direction as the LFC difference. The significance of correlation with survival was first explored on OncoLnc database (http://www.oncolnc.org/). After normalization, the expression values of the genes of interest were extracted and patients were divided into high- and low-expressing groups according to median (unless explicitly mentioned) value. Then, univariate cox proportional hazard models were computed with *surviver,* and Kaplan–Meier plots were generated with *survminer* packages, respectively. Hazard ratio (HR) and significance p-values for each gene are reported in the text and in Supplementary Table S8. All analyses for this project were done in R programming environment version 3.6 and linux operating system. All the codes are available in the Github repository.

## Supplementary Information

Supplementary Information 1.

Supplementary Information 2.
