# Supplementary material for: A pan-cancer transcriptomic study showing tumor specific alterations in central metabolism
Source: Sci Rep. 2021 Jul 1;11:13637. doi: 10.1038/s41598-021-93003-3 (PMC8249409; doi:10.1038/s41598-021-93003-3)
Supplement: Supplementary file 1 — Supplementary Information 1. [file 41598_2021_93003_MOESM1_ESM.pdf]

Supplementary Figures

A pan-cancer transcriptomic study showing tumor specific alterations in central metabolism.

Ilir Sheraj[1], N. Tulin Guray[1], Sreeparna Banerjee[1,2*]

[1]Department of Biological Sciences, Orta Dogu Teknik Universitesi (ODTU/METU), Ankara 06800, Turkey

[2] Cancer Systems Biology Laboratory (CanSyl), Orta Dogu Teknik Universitesi (ODTU/METU), Ankara 06800, Turkey.

* Corresponding author. Department of Biological Sciences, Orta Dogu Teknik Universitesi (ODTU/METU), Ankara 06800, Turkey. Email: banerjee@metu.edu.tr

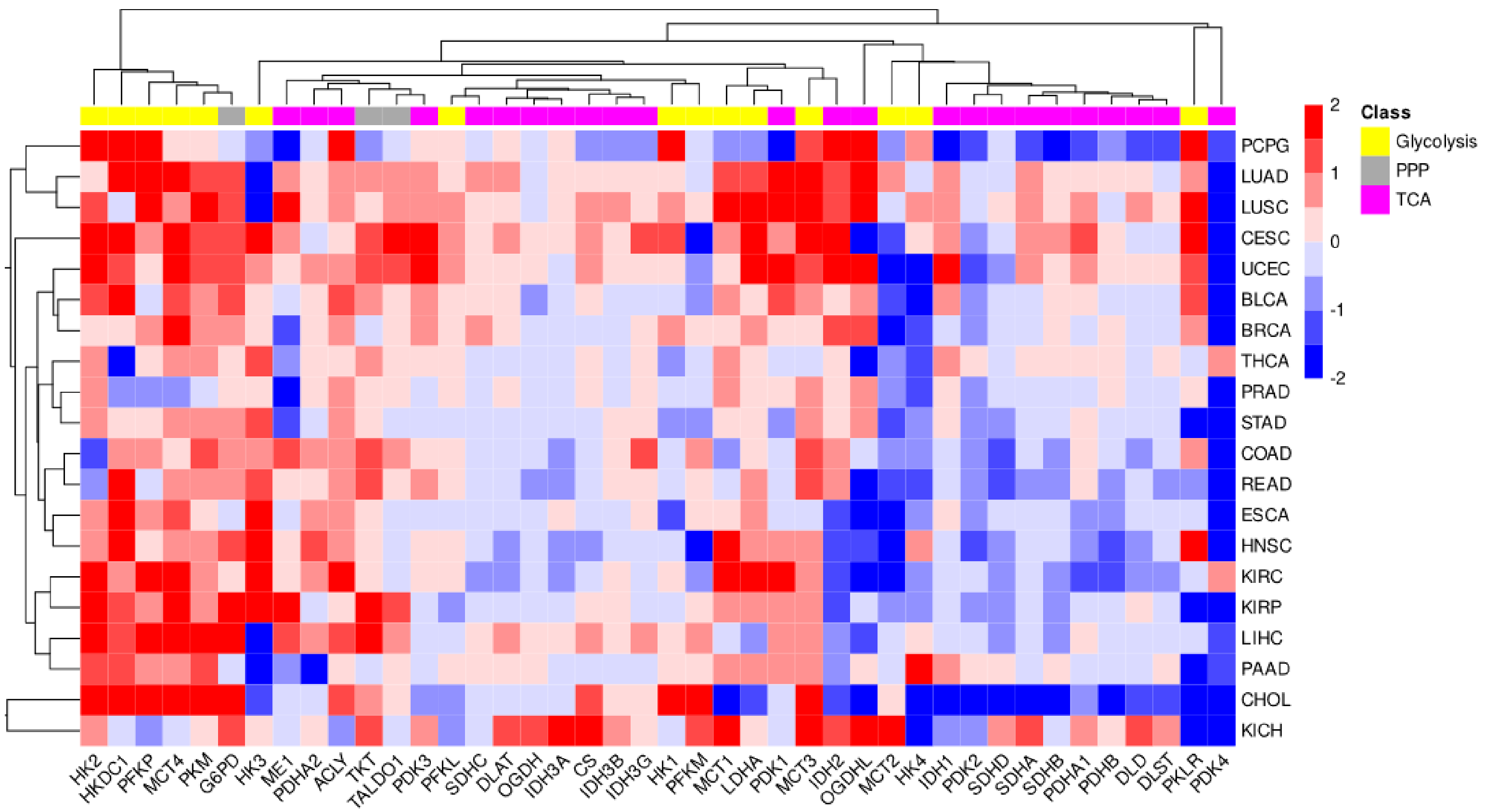

Supplementary Figure 1: Heat map showing the expression of the main enzymes controlling central metabolism of glucose. Colors in the legend show the enzymes according to their function in Glycolysis, pentose phosphate pathway (PPP) and tricarboxylic acid cycle (TCA). The color bar on the right represents the log-fold changes; red shows genes upregulated in tumors and blue shows genes downregulated in tumors as compared to matched normal samples.

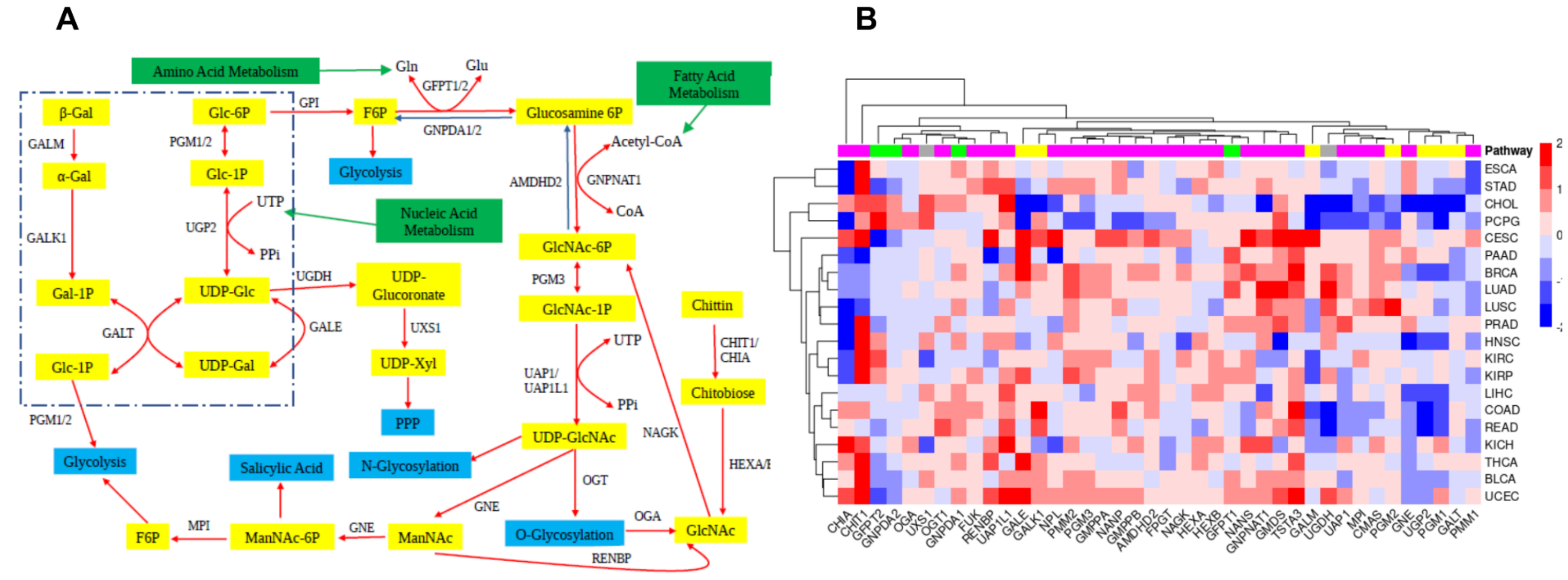

Supplementary Figure 2: **Alterations in enzymes of the hexosamine pathway between cancer and matched normal samples: (A)** Schematic representation of hexosamine pathway. Yellow color boxes show the core reactions, cyan boxes show connected pathways, and green boxes show important metabolic processes it interacts with. Two reactions requiring a separate set of enzymes to go in reverse directions are depicted by blue arrows. The Leloir pathway of galactose metabolism is enclosed in a dotted box. **(B)** Heat map showing expression of hexoasamine genes. The pathway color bar shows genes involved in Leloir pathway (yellow), hexosamine entry from glycolysis (green), UDP-glucuronate (grey) and the rest of enzymes (pink). The color bar on the right represents the log-fold changes; red shows genes upregulated in tumors and blue genes downregulated in tumors as compared to their normal counterparts. The full names of genes are found in Supplementary Table 2.

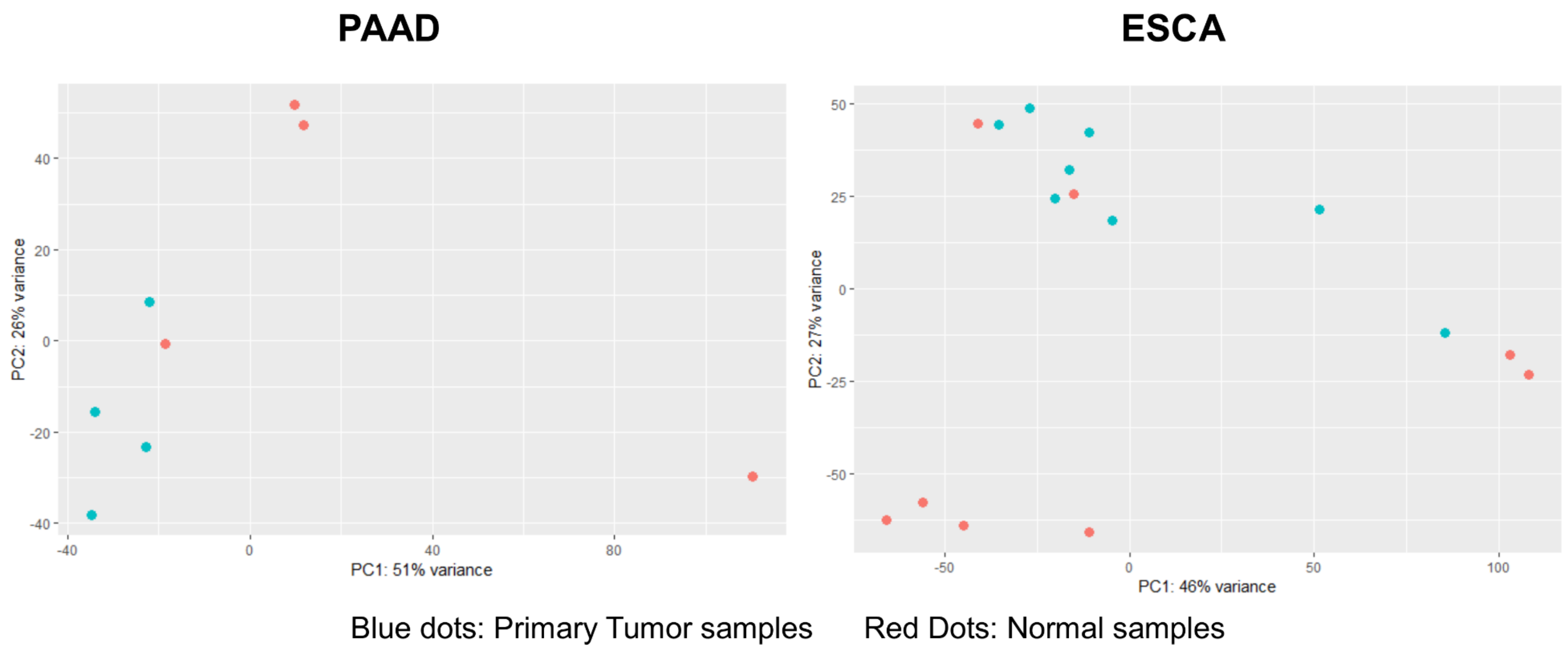

Supplementary Figure 3: Principal component analysis (PCA) plots for PAAD and ESCA. PAAD (**A**) and ESCA (**B**) showed the least number of significantly differentially expressed genes; therefore, we evaluated sample separation by PCA and found their separation to be poor. Red dots represent normal samples and blue dots their tumors counterparts. This may be the reason for very few genes showing statistical significance, despite moderate to high LFCs.

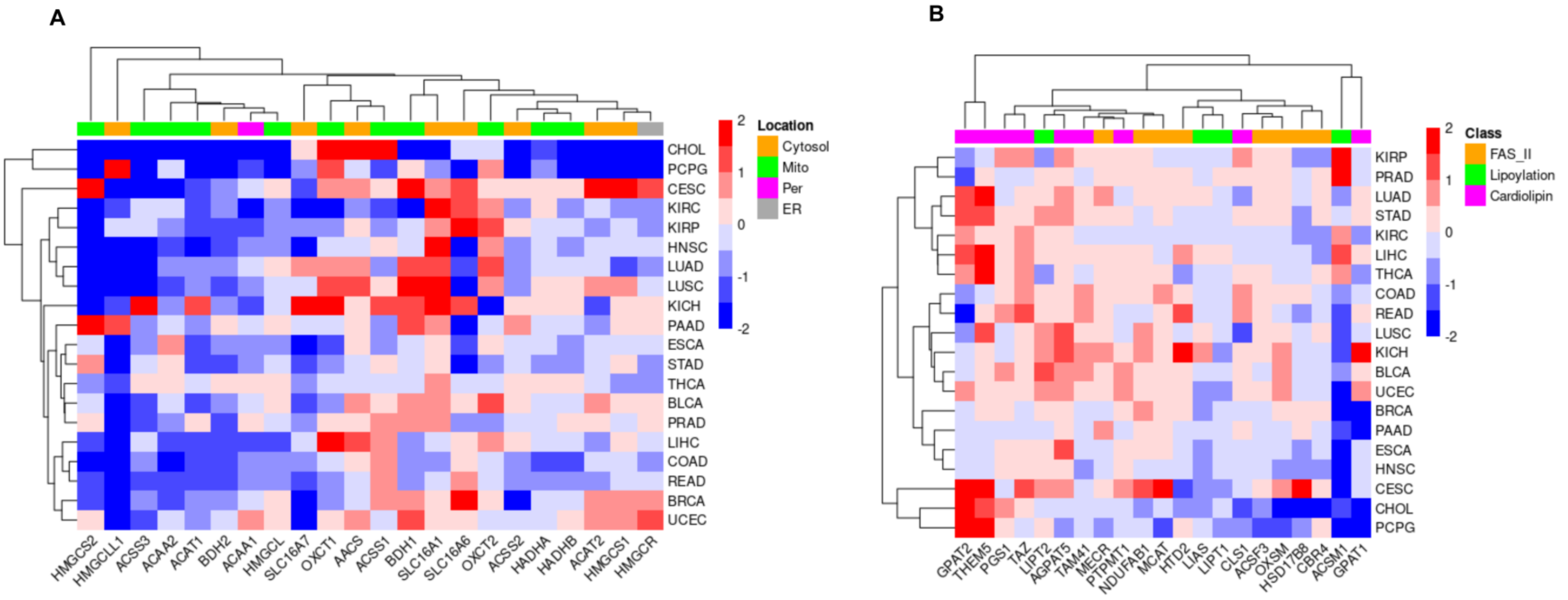

Supplementary Figure 4: Ketone Body Metabolism and Mitochondrial Fatty Acid Synthesis (FAS-II) in cancer and matched normal samples: (A) The heat map shows gene expression for hepatic ketone body synthesis and their oxidation in extrahepatic tissues. These pathways are schematically shown in Figure 3A. The color bar shows cellular localization of the enzymes in cytosol (orange), mitochondria (green), peroxisome (pink) and endoplasmic reticulum (gray). **(B)** Heat map showing enzymes involved in mitochondrial FAS. The class color bar shows enzymes involved in FAS reactions (orange), lipoylation (green) and Cardiolipin synthesis and modification (pink). The color bar on the right represents the log-fold changes; red shows genes upregulated in tumors and blue genes downregulated in tumors as compared to their normal counterparts. The full names of genes are found in Supplementary Table 3.

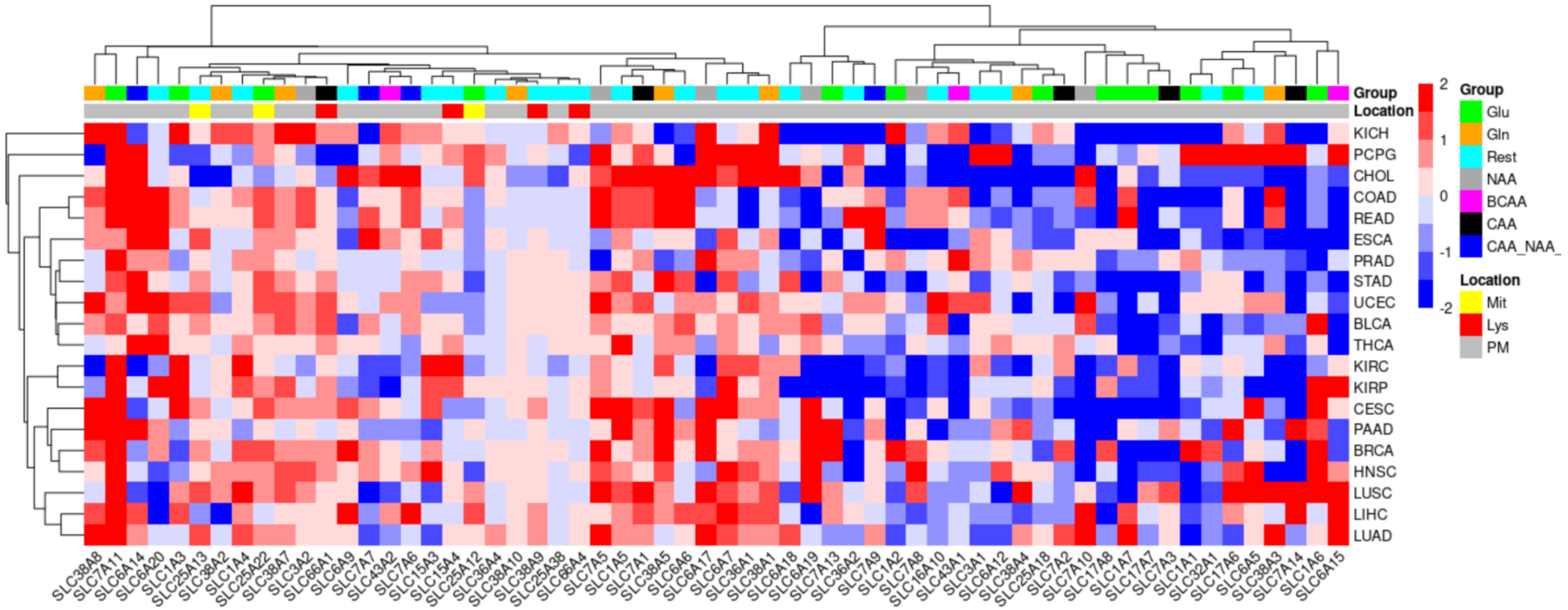

Supplementary Figure 5: **Expression of Amino Acid Transporters in cancer and matched normal samples.** Heat map showing the expression of amino acid transporters, majority of which are localized in the cellular membrane. Group color bar represents transporters involved in glutamate (green), glutamine (orange), neutral amino acids (gray), branched-chain amino acids (pink), cationic amino acids (black), both CAA and NAA (blue). The remaining category (annotated in cyan) includes many amino acids such as His, aromatic amino acids, Ala, Asp, Cys, Gly, GABA, Taurine etc. The color bar on the right represents the log-fold changes; red shows genes upregulated in tumors and blue genes downregulated in tumors as compared to their normal counterparts. For more details, please see Supplementary Table 4.

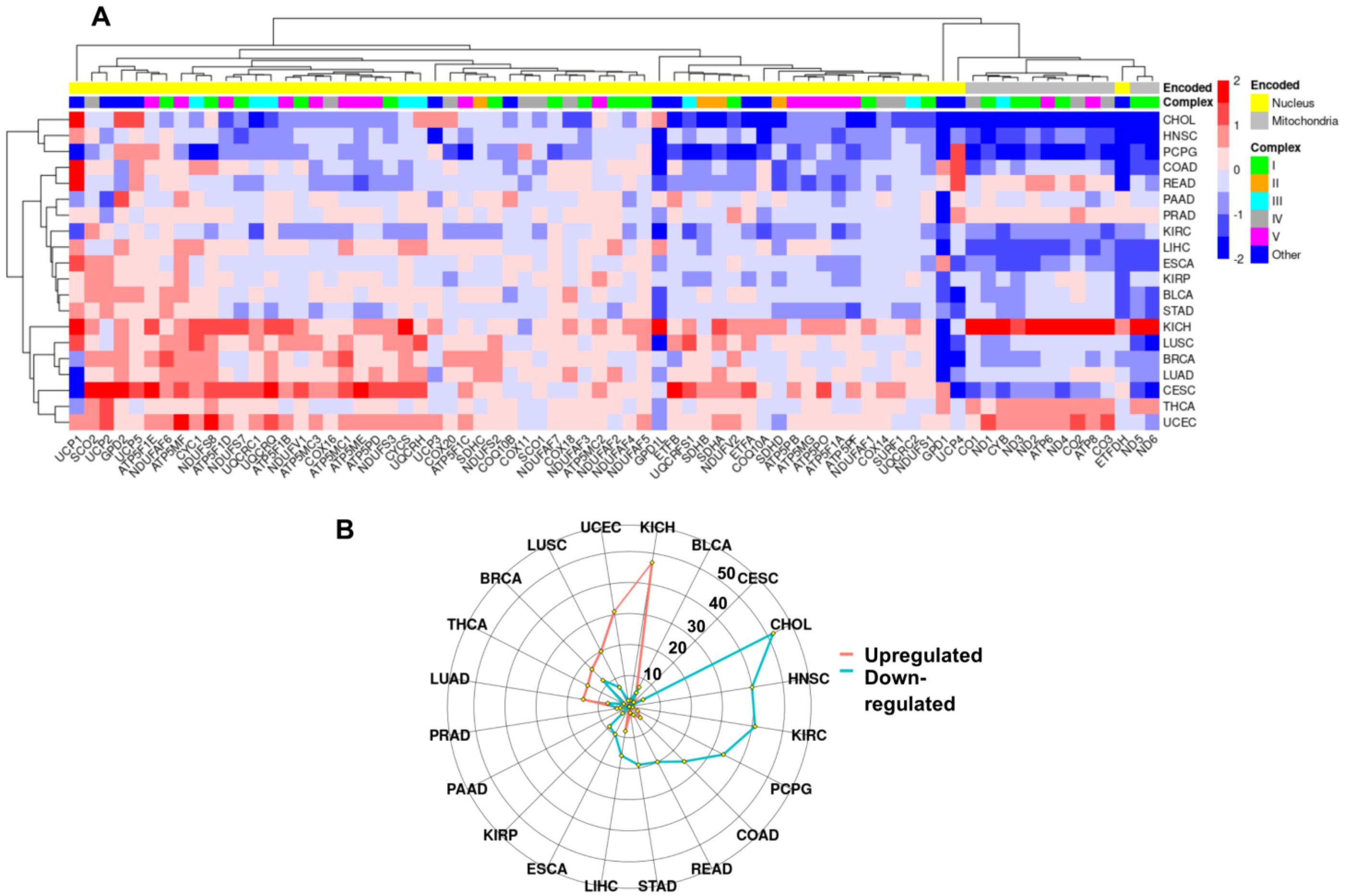

Supplementary Figure 6: Expression of Electron Transport Chain (ETC) and Glycerol 3-Phosphate Dehydrogenase (GPD) system in cancer and matched normal samples: (A) Heat map showing the expression of core enzymes involved in ETC. The Encoded bar shows whether they are encoded in nuclear genome (yellow) or mitochondrial genome (gray). Complex bar shows ETC complexes

I-IV and ATP Synthase (V). The "Other" category represents genes of the GPD system and uncoupling proteins (UCP). The color bar on the right represents the log-fold changes; red shows genes upregulated in tumors and blue genes downregulated in tumors as compared to their normal counterparts. **(B)** Radar plot showing the total number of genes significantly (LFC > 0.5, FDR < 0.05) upregulated (red) or downregulated (blue) in tumors compared to normal tissue for each cohort. The full list of genes is found in Supplementary Table 7.

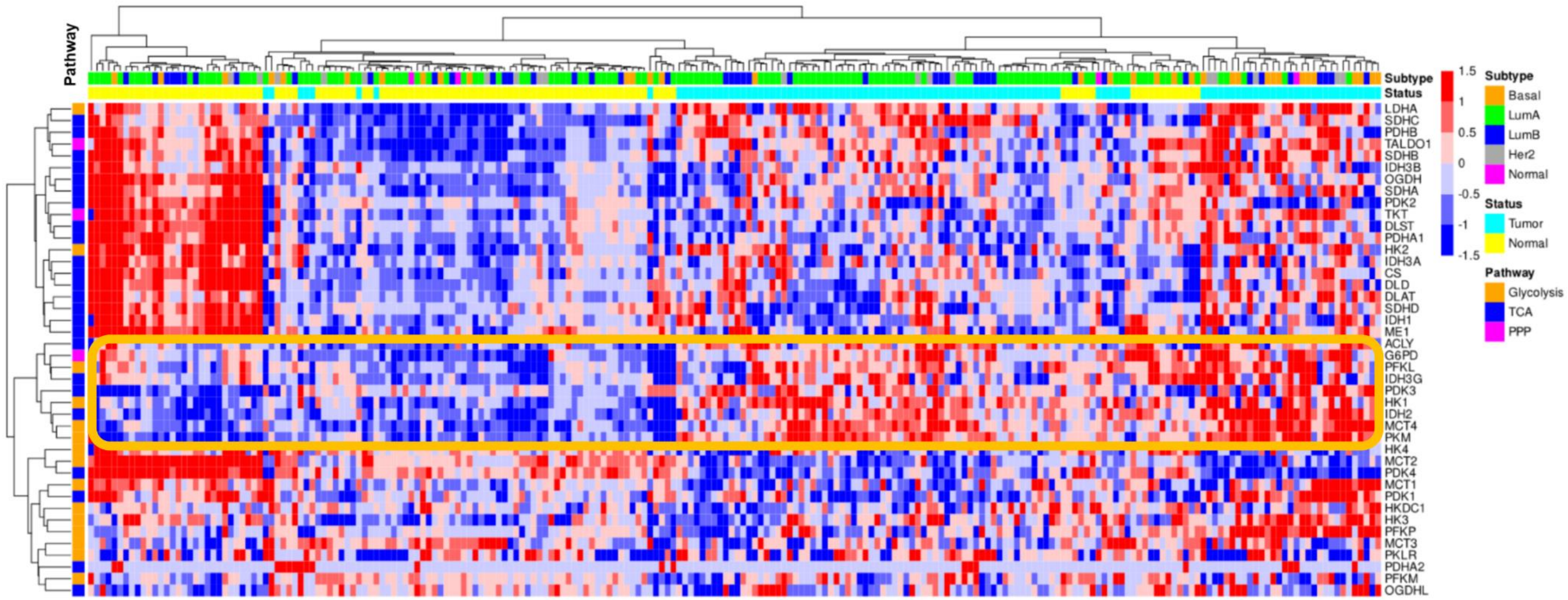

Supplementary Figure 7: Heat map showing the expression of the primary enzymes regulating glucose central metabolism in breast cancer subtypes (Basal, Luminal A, Luminal B, HER-2 and Normal like). Molecular classification of BRCA samples was obtained from Berger et al[1], and 111 tumor samples were classified into Basal (n = 17), Luminal A (n = 61), Luminal B (n = 22), HER2 (n = 9) and Normal-Like (n = 2). Vst-normalized read counts (normalized as explained in Methods) for tumor and their matched normal tissue were extracted, scaled and used to construct a heatmap. Colors in the pathway legend and under the Pathway bar show the enzymes according to their function in Glycolysis, pentose phosphate pathway (PPP) and tricarboxylic acid cycle (TCA). The Subtype bar on the top shows sample classification according to the five breast cancer subtypes. The Status bar shows sample classification into tumor and matched

normal. The LFC bar represents the log-fold changes; red shows genes upregulated blue shows genes downregulated as compared to the rest. A cluster representing the main glycolytic, PPP and Fatty Acid Biosynthesis genes is shown within the yellow box. The heat map was constructed by using pheatmap package with Euclidean distance and Ward.D2 for linkage.

References:

1 Berger, A. C. *et al.* A Comprehensive Pan-Cancer Molecular Study of Gynecologic and Breast Cancers. **33**, 690–705 (2018).
